# CICADA: An automated and flexible tool for comprehensive fMRI noise reduction

**DOI:** 10.1162/IMAG.a.114

**Published:** 2025-08-20

**Authors:** Keith Dodd, Maureen McHugo, Lauren Sarabia, Korey P. Wylie, Kristina T. Legget, Marc-Andre Cornier, Jason R. Tregellas

**Affiliations:** Department of Psychiatry, University of Colorado School of Medicine, Aurora, CO, United States; Department of Bioengineering, University of Colorado Denver, Aurora, CO, United States; Research Service, Rocky Mountain Regional VA Medical Center, Aurora, CO, United States; Division of Endocrinology, Diabetes and Metabolic Diseases, Department of Medicine, Medical University of South Carolina, Charleston, SC, United States

**Keywords:** motion, manual independent components analysis (ICA) denoising, fMRI, IC classification, FIX, ICA-AROMA

## Abstract

Independent component analysis (ICA) denoising methods can be highly effective for reducing functional magnetic resonance imaging (fMRI) noise. ICA denoising method success heavily depends, however, on the accurate classification of fMRI data ICs as either neural signal or noise. While manual IC classification (“manual ICA denoising”) is a current gold-standard, it requires extensive time and training. Automated methods of IC classification (“automated ICA denoising”), meanwhile, are less accurate and effective, especially in clinical populations where motion artifacts are more common. To address these challenges, a novel denoising method, Comprehensive Independent Component Analysis Denoising Assistant (CICADA), was developed. Uniquely, CICADA uses manual classification guidelines to automatically, comprehensively, and accurately capture most common sources of fMRI noise. As such, we hypothesized that CICADA would perform similarly to manual ICA denoising and outperform other current automated denoising methods. CICADA was evaluated against two well-established automated ICA denoising methods (FIX and ICA-AROMA) across three fMRI datasets. The datasets included high-motion resting-state (N = 57) and visual-task data (N = 53), both from individuals with schizophrenia, as well as low-motion resting-state healthy control data from an openly available dataset (N = 56). IC classification accuracy was first evaluated against manual IC classification in a subset (N = 30) of each dataset. Denoising performance efficacy was then evaluated with commonly used quality control (QC) benchmarks and correlations with fMRI noise profiles across all data. With a 97.9% mean overall accuracy in IC classification, CICADA performed nearly as well as manual IC classification and was significantly more accurate than FIX (92.9% mean overall accuracy; all p-values < 0.01) and ICA-AROMA (83.8% mean overall accuracy; all p-values < 0.001). CICADA also matched or outperformed FIX and ICA-AROMA across most QC and noise profile metrics across all data. Furthermore, CICADA greatly eased implementation of manual ICA denoising by decreasing the number of ICs a user must inspect by an average of 75%. Overall, CICADA is a novel, accurate, comprehensive, and automated ICA denoising tool for use in both resting-state and task-based fMRI. It performed similarly to the labor-intensive manual IC classification gold-standard and, in some datasets, outperformed current automated ICA denoising methods. Finally, CICADA may facilitate more efficient manual ICA denoising without reducing efficacy.

## 1 Introduction

Functional magnetic resonance imaging (fMRI) measures the blood-oxygen level dependent (BOLD) signal that corresponds with neuronal activity (Hare et al., 1998). Noise artifacts stemming from numerous sources (e.g., motion, cerebrospinal fluid [CSF] pulsations, sinus venous flow), however, may both obscure signal detection and lead to false reporting of noise as neuronal activity (Liu, 2016). While current denoising procedures include methods to reduce noise artifacts while preserving BOLD signal, some procedures may not denoise comprehensively enough, especially for particularly noisy fMRI data (Liu, 2016). As such, fMRI data with noise greater than established thresholds are often removed from analyses (Satterthwaite et al., 2013). This can lead to substantial data loss, slowing progress in psychiatric and related neuroscience research, especially for patient populations that inherently have increased noise or motion (e.g., schizophrenia) (Pardoe et al., 2016). The optimization of denoising methods is, therefore, critical for enhancing the accuracy and successful use of fMRI data, particularly for clinical populations.

Independent component analysis (ICA) methods currently have the greatest potential to reduce noise artifacts while retaining BOLD signal across many fMRI paradigms (Ciric et al., 2017; Parkes et al., 2018; Pruim, Mennes, Buitelaar, et al., 2015). ICA denoising methods accomplish this by first decomposing fMRI data into spatially independent components (ICs). Next, each IC is classified as neural signal or noise, by either manual, semi-automated (e.g., a subset of manually classified ICs trains a model to automate the rest of the classification), or automated methods. Finally, the ICs labeled as noise are regressed out of the data. Of note, the regression model may include only the noise ICs (“aggressive denoising”), or both the signal and noise ICs (“non-aggressive denoising”) (Pruim, Mennes, van Rooij, et al., 2015). If aggressive denoising is applied, noise removal is maximized. If non-aggressive denoising is applied instead, retention of BOLD signal and functional connectivity (FC) is prioritized while still reducing noise components (Griffanti et al., 2017).

Manual classification of IC components by experts remains a gold standard in ICA denoising (Griffanti et al., 2017). Manual ICA denoising, however, is time-consuming, introduces subjective signal and noise IC classification, and is complicated by the need for training to develop expertise. Semi-automated or automated IC classification methods, such as FMRIB’s ICA-based Xnoiseifier (FIX – semi-automated) (Salimi-Khorshidi et al., 2014), ICA Automatic Removal of Motion Artifact (ICA-AROMA – fully automated) (Pruim, Mennes, van Rooij, et al., 2015), and Alternative Labeling Tool (ALT – fully automated) (Zhukovsky et al., 2022), help ameliorate manual classification limitations. These methods have their own limitations, however. To our knowledge, there are currently no fully automated methods that effectively target all the most common sources of fMRI noise (i.e., motion, physiologic, and hardware noise). For example, ICA-AROMA is designed to accurately and specifically target motion-related noise, while ALT non-specifically identifies some aspects of various noise artifacts. Meanwhile, semi-automated methods, such as FIX, may better target more noise sources, but they still require a substantial amount of initial manual IC classification to train the model. As such, the development of an ICA denoising method that automatically and accurately classifies all common sources of fMRI noise has high potential to improve denoising efficiency.

In the current work, we propose a novel automated ICA-based denoising method, Comprehensive ICA Denoising Assistant (CICADA). CICADA addresses the limitations of other ICA denoising methods by fully automating manual IC classification guidelines to target all common sources of fMRI noise (Griffanti et al., 2017). This work first describes how CICADA uses IC spatial maps, timeseries, and power spectra to classify ICs as signal or different types of noise. Next, CICADA is compared to other widely-adopted automated ICA-based methods (FIX, ICA-AROMA) across three datasets with varying paradigms and levels of motion. Overall, CICADA was compared to FIX and ICA-AROMA in several complementary analyses. First, IC classification accuracy was compared to manual IC classification. Second, denoising performance was compared to CICADA with established Quality Control (QC) benchmarks, including motion-FC correlations and distance dependence. Finally, denoising performance was compared to CICADA by a novel method referred to as “noise profiles”, including a new “Denoising Success” parameter. The three datasets used to evaluate CICADA included two high-motion datasets (one resting-state and one task-based) from individuals with schizophrenia and a low-motion resting-state dataset from healthy controls.

As CICADA was designed to capture more sources of fMRI noise than ICA-AROMA, we hypothesized that CICADA would improve data quality more than ICA-AROMA. In contrast, as CICADA was designed to automate the manual processes involved in manual ICA denoising and the semi-automated FIX, we also hypothesized that CICADA would improve the data quality similarly to these methods. With more comprehensive denoising than other fully automated techniques, CICADA may allow for more efficient fMRI denoising with efficacy approaching that of the manual gold-standard.

## 2 Methods

**Fig. 1.**
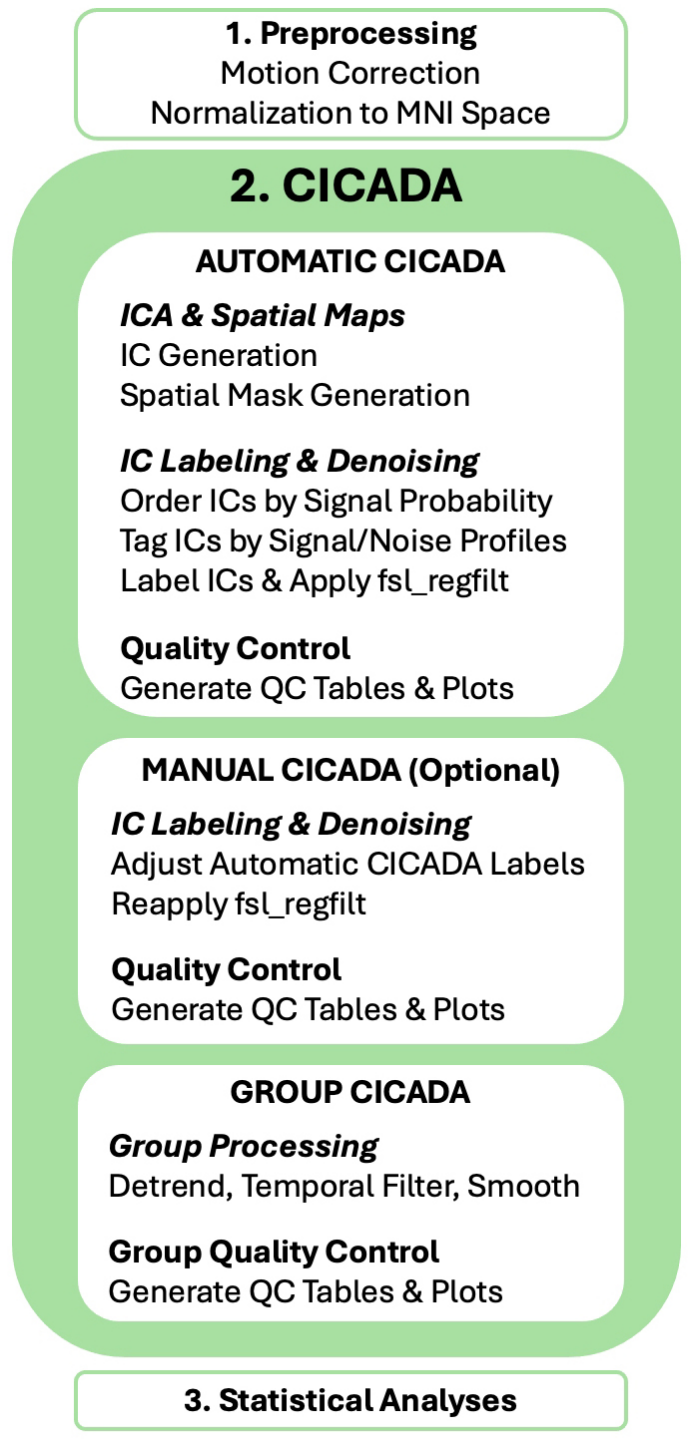
CICADA Pipeline.

CICADA is designed to follow standard preprocessing pipelines (e.g., fMRIPrep (Esteban et al., 2018)), including motion correction and normalization, with the exception of spatial smoothing as CICADA spatially smooths at the end of its pipeline. CICADA is relatively simple to use and install, with only Matlab and FSL as requirements. CICADA is freely available on GitHub (https://github.com/keithcdodd/CICADA), where a user guide and example scripts (“example_CICADA_flow”) are included. It does not require training data or parameter tuning and can be used for both resting-state and task-based fMRI data without modification. CICADA can be subdivided into three major functions, Automatic, Manual, and Group CICADA (Fig. 1), each described in detail below.

### 2.1 Automatic CICADA

**Fig. 2.**
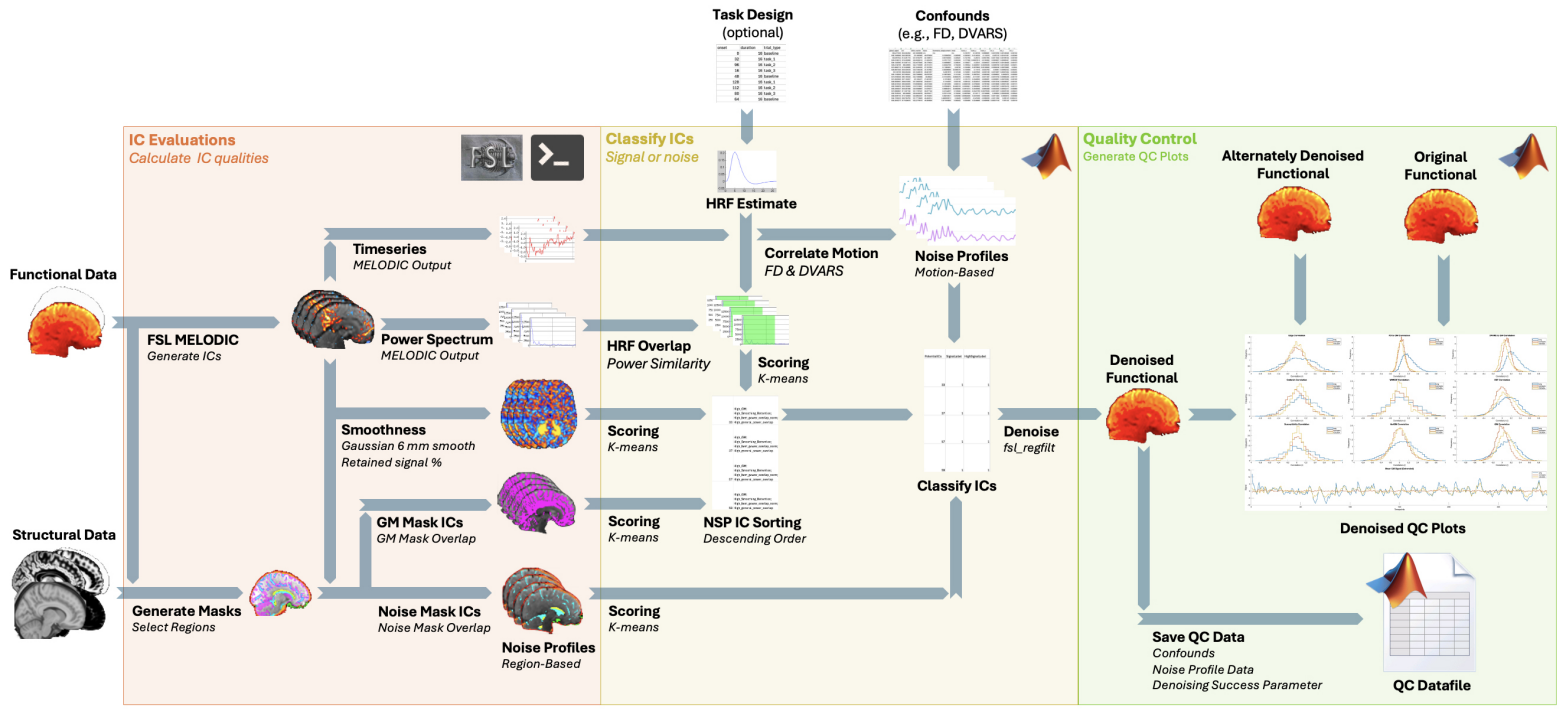
Schematic of the Automatic CICADA Pipeline. Required inputs for the Automatic CICADA Pipeline include an fMRI file with a binary functional brain extracted mask (“Functional Data”) and a confounds file (“Confounds”). Other inputs include structural MRI data (“Structural Data”: e.g., T1, binary structural brain extracted mask, and tissue segmentation—gray matter, white matter, cerebral spinal fluid—probability files), and a task design file for task fMRI (“Task Design”). Relevant Abbreviations: ICs: independent components. NSP: Neural Signal Probability.

Automatic CICADA aims to automate application of manual IC classification guidelines, as described in Griffanti et al. (2017). ICs can be classified as signal or noise based on their spatial maps, timeseries, and power spectra. For example, neural signal-like ICs are typically characterized by the following features: (1) a smooth spatial map that covers mostly gray matter; (2) a timeseries with limited correlations to movement; and (3) a low frequency (e.g., 0.008–0.15 Hz) power spectrum resembling that of expected BOLD hemodynamic responses. Noise-like ICs, meanwhile, have their own unique spatial maps, timeseries correlations, and power spectra, depending on the dominating source of the noise. Motion-dominant ICs, for example, are often characterized by the following features: (1) a spatial map concentrated to the edge of the functional image; (2) a timeseries highly correlated to motion parameters; and (3) a power spectrum containing higher frequencies (e.g., >0.15 Hz) that are uncommon in BOLD hemodynamic responses. Altogether, Automatic CICADA uses these features to automatically differentiate neural signal from common noise sources in a manner similar to the manual IC classification guidelines (Griffanti et al., 2017). A general schematic of the Automatic CICADA Pipeline is provided in Figure 2, with more details described below.

#### 2.1.1 CICADA independent component evaluations

**Fig. 3.**
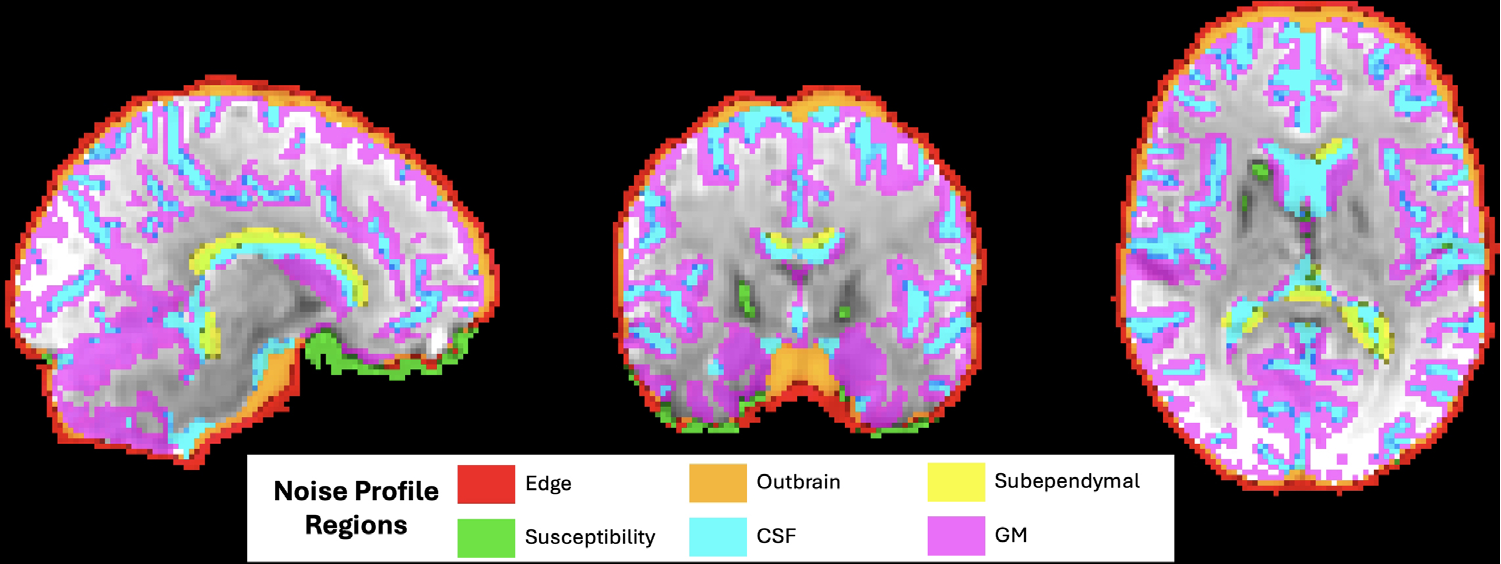
CICADA-Generated Region Masks Example. This figure displays a functional MR image with CICADA-generated region masks that help characterize commonly seen signal and noise components.

First, Automatic CICADA runs MELODIC (Beckmann & Smith, 2004) with default parameters to obtain spatial maps, timeseries, and power spectra of ICs. Next, CICADA creates subject-specific masks (“region masks”) that encompass regions strongly associated with common signal and noise components (Fig. 3) (Griffanti et al., 2017). In short, these region masks are generated through use of the following reference files: an anatomical mask, a functional mask, the functional file itself, and gray matter (GM), white matter (WM), and cerebral spinal fluid (CSF) probability files (see Supplementary Material on “basescript 1” for more information). The associated regions for the region masks include gray matter (“GM”), the edge of the functional mask (“edge”), the meninges and meningeal spaces (“outbrain”), subependymal veins (“subependymal”), cerebrospinal fluid (“CSF”), and susceptibility-impacted regions (“susceptibility”). All other regions within the functional mask but outside the GM are labeled as “notGM”. These region masks are then used to calculate the degree of spatial overlap of each IC map with each region mask (“regional spatial overlap”). Briefly, for each region mask and IC, regional spatial overlap is calculated by summing the magnitude of the non-thresholded IC spatial map after applying the region mask.

Next, Automatic CICADA calculates motion-related IC timeseries correlations (“motion correlations”), spatial map smoothness (“smoothness”), and overlap of IC power spectra with estimated hemodynamic response function (HRF) power spectra (“power spectrum overlap”). Motion correlations are calculated as the correlation between motion-related parameters (framewise displacement [FD] (Power et al., 2012) and temporal Derivative of root mean square VARiance over voxels [DVARS] (Power et al., 2012; Smyser et al., 2011)) and the derivative magnitude of the IC timeseries. The derivative magnitude was selected because both FD and DVARS are mathematically derivative magnitudes of measurements over time. Of note, FD was calculated using the formula proposed by Power et al. (2012). Spatial map smoothness is estimated as the ratio of retained signal of a non-thresholded IC z-stat map following 6 mm Gaussian smoothing (see Supplementary Material on “basescript 1” and “basescript 2”). Power spectrum overlap for each IC is calculated by multiplying, and then summating, an estimated hemodynamic response function (HRF) power spectra by the IC power spectra. For resting-state and task-based designs, a single HRF response is estimated using a double gamma function (Friston et al., 1994). Additionally, if a task design is provided to CICADA, double gamma HRFs are also estimated both for each trial-type and across all trials. Hereafter, “IC evaluations” refers to all these measurements (regional spatial overlap, motion correlations, smoothness, and power spectrum overlap).

#### 2.1.2 CICADA IC classification

As previously described, neural signal-like ICs should be characterized by higher GM spatial overlap, spatial smoothness, and power spectrum overlap with estimated BOLD hemodynamic responses. Similarly, all other IC evaluations in neural signal-like ICs should be relatively lower compared to most other noise-like ICs. Therefore, to assist in delineating between neural signal-like and noise-like ICs, CICADA categorizes the evaluations for each IC as either “high”, “average”, or “low” in value. This categorization is accomplished through a standard k-means three-group clustering algorithm for each IC evaluation across all ICs (referred to hereon as “k-means categorization”). Automatic CICADA then sorts the ICs from high relative neural signal probability (NSP) to low NSP, based on the following equation:

$$NSP=norm\left(S\right)*norm{\left(GMO\right)}^{2}*norm\left(PSO\right),$$

where S is smoothness, GMO is gray matter spatial overlap, and PSO is power spectrum overlap. In each case, “norm” refers to a normalization of each parameter by rescaling the range to [0,1]. For task-based scans, the highest PSO (the single HRF estimate or an HRF estimate for the task design) is used in the equation. Overall, this equation takes advantage of the higher S, GMO, and PSO of neural signal dominant ICs. As GMO is likely more indicative of neural signal than either S or PSO (e.g., subependymal noise has high S and PSO, but lower GMO), GMO is squared to increase the weight of its value.

The ICs are sorted from high to low NSP, then classified as either signal or noise. To accomplish this, CICADA evaluates the ICs in NSP order, and classifies an IC as signal if all the following criteria are met:

1. The IC is k-means classified as high in either GMO, PSO, or S.
2. The IC is either k-means classified as high in GMO or is not high in any other regional spatial overlap.
3. The IC either has no noise-like k-means labels or is k-means classified as high in both GMO and either PSO or S.

Importantly, these three criteria aim to follow the standard mantra of “innocent until proven guilty.” Altogether, little evidence of signal is required to be considered for classification as signal (criterion 1), and even with strong evidence of noise, concurrent evidence of signal takes priority (criteria 2 and 3). Notably, by following these three criteria, Automatic CICADA uses all calculated IC evaluations (not only GMO, PSO, S) to decide on signal or noise IC classifications. Furthermore, by examining the ICs in NSP order, Automatic CICADA can take advantage of a “tolerance value” to improve efficiency. More specifically, Automatic CICADA stops inspecting ICs when either the tolerance value (set to 5 by default and decreasing each time an IC is classified as noise-like) is reduced to 0, or when the current IC NSP falls below the mean IC NSP. The default tolerance value of 5 was chosen as, upon testing, a higher value did not improve performance on the datasets (see Supplementary Material 1.3.2 for more details on the tolerance value). Every IC not examined is then also classified as noise. Overall, this automated IC classification approach is designed to align with the manual IC classification guidelines provided in Griffanti et al. (2017).

The results of the classification, alongside the k-means categorizations, are recorded in an “IC Checker” spreadsheet (see Supplementary Fig. 3 and Supplementary Material 1.4). The IC Checker also includes k-means categorizations of IC spatial overlap with common functional networks to assist in network identification and avoid excluding data of interest. This is accomplished in the same manner as regional spatial overlap but with masks from a seven-network atlas encompassing the entire brain (Guzmán-Vélez et al., 2022; Yeo et al., 2011). Automatic CICADA then performs non-aggressive regression by default (including both signal and noise ICs in the model (Pruim, Mennes, van Rooij, et al., 2015)) to remove noise components using FSL’s fsl_regfilt function. If desired, CICADA can apply aggressive regression instead. Automatic CICADA also separately performs eight parameter denoising (aggressive [i.e., standard] regression of the six motion parameters + mean white matter [WM] timeseries + mean CSF timeseries) for simple comparison during quality control.

#### 2.1.3 CICADA quality control

Finally, Automatic CICADA performs subject-level quality control (QC) analyses and compares the denoised data to the original data (before denoising), as well as to comparison data (eight parameter denoised). QC measurements include the number, and percentage, of ICs that were classified as signal. The percent signal variance retained by CICADA is also estimated by summating the percent signal variance (as output by MELODIC) of each IC retained by CICADA. Automatic CICADA also produces images of voxel-wise connectivity to seeds of seven major brain networks before and after denoising (see Supplementary Material 1.3.3). Furthermore, Automatic CICADA plots a set of novel QC metrics termed “noise profiles.” These metrics can be characterized as two types: region-based and confound-based noise profiles.

A region-based noise profile refers to the distribution of correlations between the timeseries of randomly sampled pairs of voxels within regions identified as common sources of noise in manual IC classification. These regions include edge, outbrain, subependymal, CSF, susceptibility, and notGM. For region-based noise profiles, CICADA randomly samples 10,000 voxels and correlates timeseries, giving 49,995,000 uniquely sampled pairs. In cases where a given region may not contain 10,000 voxels (i.e., subependymal space), all voxel pairs within the region are used.

Confound-based noise profiles, meanwhile, refer to correlations between each of two confound parameters (FD and DVARS) and the derivative magnitude of randomly sampled voxel timeseries (10,000 voxels) within the GM region mask. Since both FD and DVARS parameters are heavily associated with head movement, the confound-based noise profiles are highly reflective of motion and referred to as FD-GM and DVARS-GM noise profiles hereon.

Importantly, noise-induced connectivity (i.e., voxel pair correlations) may manifest as either negative or positive correlations. For example, motion, alongside spatial resampling, has been shown to induce both positive and negative voxel correlations (Yuan et al., 2016). As such, the overall strength (instead of sign) of correlations for each noise profile is most indicative of retained noise. Therefore, for every noise profile, reduction of noise is characterized by a reduced mean magnitude of the corresponding noise profile correlations.

In addition to plotting the six region-based and two confound-based noise profiles, Automatic CICADA also displays GM profile correlations (calculated in the same manner as the region-based noise profiles) and the mean GM signal timeseries for reference (see Supplementary Fig. 4). Importantly, GM profile correlations are not considered a noise profile metric by themselves but instead are used as reference for comparison with the noise profiles. This is necessary since successful ICA denoising will typically retain correlations between voxels preferentially in the GM as opposed to outside the GM (i.e., within region-based noise profiles). Therefore, since GM correlations are mainly positive (Schölvinck et al., 2010), a GM profile correlation that is more positively skewed than the notGM profile correlation may suggest that noise, as opposed to signal, was preferentially reduced. In total, Automatic CICADA generates 10 quality control plots comparing the Automatic CICADA denoised data to the comparison and original images.

### 2.2 Manual CICADA

Manual CICADA is an optional step that allows the user to perform manual ICA denoising in an expedited fashion. If implemented, Manual CICADA occurs after Automatic CICADA but before Group CICADA. In short, the only required manual step of Manual CICADA is adjustment of the IC classifications in the “IC Checker” spreadsheet (see section 2.1.2) provided by Automatic CICADA. From there, Manual CICADA performs the same methods as Automatic CICADA, starting with the regression (non-aggressive by default) of the noise-labeled ICs. The IC Checker’s NSP IC ordering, alongside the k-means categorizations, aims to both greatly reduce the number of ICs a user must examine, and to assist with IC classification decision making.

### 2.3 Group CICADA

Group CICADA is designed to prepare data for subsequent statistical analyses. Importantly, Group CICADA serves mainly as a post-denoising quality control step. It aggregates the data, conducts additional preprocessing steps, and calculates group-level QC. First, Group CICADA combines subject-level QC data, such as the percent of ICs labeled as signal, the dice coefficient between the signal-labeled IC spatial maps and the GM mask, and subject-level flagging of image outliers. Several methods of outlier detection are provided, including conservative and liberal motion thresholds derived from Satterthwaite et al. (2013), as well as a new “CICADA outlier” detection method. In short, CICADA outliers are functional images in which CICADA was unable to reliably find and retain signal-dominant ICs (see Supplementary Material section 1.5.1). Next, Group CICADA further preprocesses the data by performing optional detrending (2^nd^ order polynomial default), bandpass temporal filtering (no bandpass default), gaussian smoothing (FWHM = 1.5 * voxel size default), and voxel-wise intensity normalization (no intensity normalization default). These settings can all be customized by the user.

Finally, Group CICADA generates group-level QC plots, performs group-level IC decomposition with FSL’s MELODIC, and estimates which group-level ICs best match each of seven major network masks (Guzmán-Vélez et al., 2022; Yeo et al., 2011). This matching is performed using a greedy Dice-based approach: each IC’s spatial probability map is thresholded at ≥99% probability to create a binary mask, and for each network, ICs are iteratively selected to maximize spatial Dice overlap with the network mask. The process stops when three consecutive ICs fail to improve overlap. This final identification of ICs matching the seven major networks acts as a final QC check: well-denoised data should retain group-level ICs that correspond well with known functional networks.

### 2.4 MRI datasets

To compare its effectiveness in a variety of fMRI data, CICADA was evaluated across three datasets, including high-motion resting-state data (“high-motion rest”; N = 57, group-level median framewise displacement [mFD] = 0.20 mm), high-motion visual-task data (“high-motion task”; N = 53, group-level mFD = 0.26 mm), and low-motion resting-state data (“low-motion rest”; N = 56, group-level mFD = 0.09 mm). Of note, group-level mFD is calculated as the median of the subject-level median framewise displacement. Both high-motion task-based and resting-state data were collected during a single scanning session from individuals with schizophrenia or schizoaffective disorder, as assessed by the Structured Clinical Interview for DSM-V (First et al., 2016). These data were part of a larger study investigating the effects of neuroleptics and exercise on the brain response to visual food cues (in both fasted and fed states) in individuals with schizophrenia (registered at clinicaltrials.gov as NCT02455193). All participants provided written informed consent. Furthermore, all procedures were in accordance with and approved by the Colorado Multiple Institutional Review Board. The current analyses utilized only baseline measures from participants in this larger study during their first scan, which was completed after an overnight fast (≥10 h). Four participants did not complete a resting-state scan following the task-based scan. One participant’s task-based scan was removed from analyses after manual IC classification confirmed that no ICs met criteria for identifiable neural signal. As such, sample sizes for task-based and resting-state scans were not equivalent.

For the low-motion dataset, the resting-state data were part of the openly-available Human Connectome Project for Early Psychosis (HCP-EP) (Levitt et al., 2023). The subset included here were classified as “healthy controls” in the HCP-EP dataset (N = 56, mFD = 0.09 mm). Per HCP-EP “healthy control” criteria, these participants were not treated with psychiatric medications at the time of study entry, did not meet criteria for a history of DSM-V diagnosis (although a >1 year anxiety disorder duration was allowable if in remission for at least 12 months without medication), had no history of psychiatric hospitalization, and reported no first-degree family members with a schizophrenia spectrum disorder. All available HCP-EP control subjects were included in the analyses to approximate the sample size in the high-motion datasets (see Table 2). The group-level mFD was less than half that of the high-motion datasets (mFD = 0.09, see Table 2).

### 2.5 MRI acquisition

The high-motion fMRI datasets were acquired on a Siemens Skyra 3T scanner equipped with a 20-channel head coil at the University of Colorado Anschutz Medical Campus Brain Imaging Center. Imaging included a T1-weighted structural scan (slice thickness = 0.9 mm, repetition time [TR] = 2300 ms, echo time [TE] = 2 ms) and two gradient-echo T2* scans (slice thickness = 2.6 mm, gap = 1.4 mm, TE = 30 ms, TR = 2000 ms, resting-state scan duration = 600 s, task-based scan duration = 344 s).

The low-motion MRI data (from HCP-EP) were acquired on Siemens MAGNETOM Prisma 3T scanners with a multiband sequence and 32/64-channel head coils. Imaging included a T1-weighted structural scan (isotropic resolution = 0.8 mm, TR = 2400 ms, TE = 2.22 ms) and a resting-state scan (voxel size = 2 mm^3^, TR = 729 ms, TE = 37 ms, multiband acceleration factor = 8, duration = 328 s). Only the functional images with posterior-anterior phase encoding were used for the current work.

### 2.6 Data preprocessing

All data were preprocessed with fMRIPrep v23.1.4 (Esteban et al., 2018). Briefly, steps included motion correction and normalization to the Montreal Neurological Institute (MNI) 2009c nonlinear asymmetric T1-weighted template. Only the high-motion datasets included slice timing due to the longer TR. Low-motion resting-state data were corrected for intensity non-uniformity. The fMRIPrep outputs in MNI 2009c space were used as the inputs for all evaluated denoising methods.

### 2.7 Denoising pipelines

The primary aim of this work was to evaluate CICADA’s performance against manual denoising and the most widely used IC denoisers. Therefore, all data were denoised by CICADA, FIX (v1.06.15) (Griffanti et al., 2014; Salimi-Khorshidi et al., 2014), and ICA-AROMA (v0.4.4) (Pruim, Mennes, van Rooij, et al., 2015). For all methods, the same MELODIC ICs were used. To standardize analytic preprocessing pipelines, and ensure the same ICs were compared across all methods, data were not smoothed prior to denoising. Additionally, data were only detrended to the second polynomial (not temporally filtered, spatially smoothed, or intensity normalized) across all denoising methods, including CICADA, to better focus on the effects of IC classification differences. Automatic CICADA was implemented as described above, with the other denoising methods described in more detail below.

#### 2.7.1 Manual ICA denoising

Manual ICA denoising was performed following the standard guidelines in Griffanti et al. (2017). Given that IC classification decisions may not always be clear, manual raters will individually vary in their classification decisions. Therefore, the final manual IC classification was reached through consensus of multiple raters. Specifically, ICs were hand-labeled as signal or noise by authors KD and LS independently, with discrepancies independently resolved by author MM. Of note, neither LS nor MM were involved in developing CICADA’s methods, thereby minimizing potential bias in manual IC classification decisions. Manual denoising was applied to a subset of N = 30 in each dataset (“manual subsets”), matching the manual subset size of other similar work (Pruim, Mennes, van Rooij, et al., 2015; Zhukovsky et al., 2022). Following manual IC classification, data were denoised using non-aggressive regression (Pruim, Mennes, van Rooij, et al., 2015) and processed with the Automatic CICADA QC (section 2.1.3) and Group CICADA (section 2.3) pipelines. Hereafter, “manual IC” refers to these final manual IC classification group decisions and subsequent denoising. Meanwhile, “individual rater” (“manual individual rater classification”: MIRC) refers to the independent and individual manual IC classification decisions by KD and LS.

#### 2.7.2 Manual individual rater classification (MIRC)

Consensus between multiple raters in manual denoising increases IC classification accuracy but is challenging to implement due to time and training constraints. Thus, manual ICA denoising is often performed by a single manual rater. Therefore, it is important to compare the IC classification of CICADA not only to the final IC classification consensus but also to the manual individual rater classification. In the current work, therefore, MIRC IC classification accuracy (see section 2.8.1) refers to the mean IC classification accuracy (see section 2.8.1) of the individual raters, as compared to the final manual IC classification group consensus.

#### 2.7.3 FIX

FIX (version 1.06.15) is a semi-automated ICA denoising technique designed to replicate manual ICA denoising without the need to manually classify every image (Griffanti et al., 2014; Salimi-Khorshidi et al., 2014). In short, manual IC labeling on a subset of data is used to train the feature-rich FIX model, which then classifies and denoises the rest of the data. In the current work, ICs from 10 subjects (the minimum suggested by FIX (Salimi-Khorshidi et al., 2014)) in each dataset were manually classified (by KD) to train the FIX model before applying it to the rest of the data. To minimize FIX bias, the 30-subject manual subsets used for manual ICA denoising did not include the subjects that were used to train the FIX classifier. To maintain consistency across methods, the “-m” flag, to optionally also regress motion confounds, was not used.

#### 2.7.4 ICA-AROMA

ICA-AROMA (version 0.4.4) is a fully automated ICA-based denoising technique intended to specifically remove motion-related noise (Pruim, Mennes, van Rooij, et al., 2015). In short, ICA-AROMA uses IC spatial overlap with both edge and CSF masks, IC timeseries correlations to motion parameters, and the proportion of the power spectra contained in higher frequencies to categorize each IC as either motion or non-motion. ICA-AROMA then applies nonaggressive regression of the motion ICs. In this work, the standard ICA-AROMA function was applied to all data. Of note, while pre-smoothing the data is common in ICA-AROMA, this was not applied to remain consistent in comparing the various methods.

### 2.8 Denoising performance measures

Denoising performance measures addressed two primary aims: (1) to determine how IC classification accuracy of CICADA compares to other ICA denoising methods and (2) to determine how well CICADA denoises fMRI data compared to other methods. To this end, IC classification accuracy of the ICA denoising methods (CICADA, FIX, and ICA-AROMA) as well as MIRC (mean inter-rater classification accuracy, section 2.7.2) were first evaluated in the manual subsets. Second, QC measures of automatically denoised data (by CICADA, FIX, ICA-AROMA) as well as the original preprocessed data before denoising (“original data”) were evaluated.

Statistical significance for each denoising performance measure was evaluated through paired nonparametric tests (Wilcoxon signed rank (Wilcoxon, 1945)) between CICADA and each other denoising method unless otherwise specified. Tests were considered significant if they survived Bonferroni correction at the p = 0.05 level for each method comparison. Classification accuracy p-values were corrected for three tests (MIRC, FIX, ICA-AROMA; adjusted p = 0.0167). All other comparisons were also corrected for three tests (FIX, ICA-AROMA, original data; adjusted p = 0.0167). The denoising performance measures are described in more detail below.

#### 2.8.1 IC classification accuracy

For each subject, IC classification from each method was compared against the “ground truth” of manual IC classification. Altogether, we used six measures of accuracy to evaluate IC classification performance based on a confusion matrix: noise sensitivity (NS; equivalent to signal specificity), noise predictive value (NPV), signal sensitivity (SS; equivalent to noise specificity), signal predictive value (SPV), overall accuracy (OA), and the signal F1 score (F_S_). Noise and signal sensitivities represent the proportion of manually-classified noise or signal ICs, respectively, that are correctly classified by the denoising method. Meanwhile, noise and signal predictive values are the proportion of method-classified noise or signal ICs, respectively, that are correctly classified. Overall accuracy indicates the total proportion of ICs classified correctly by the denoising method. Given the low prevalence of signal ICs, however, this measure may be misleading. As such, a signal F1 score, the harmonic mean of signal predictive value and signal sensitivity, may more appropriately quantify overall IC classification success. These measures are explained in more detail in the Supplementary Material (section 2.1).

Accuracy significance was determined by signed rank tests of the subject-level mean accuracy differences between Automatic CICADA and each of the other IC classification methods (FIX and ICA-AROMA), as well as the manual individual rater classification accuracy. Significantly greater accuracy parameters indicate increased accuracy as compared to CICADA IC classification.

#### 2.8.2 Manual IC classification efficiency

Projected efficiency of manual IC classification implementation following Automatic CICADA (i.e., Manual CICADA) was quantified through the mean and maximum percentile rank of the last manually labeled signal IC following Automatic CICADA’s relative neural signal probability IC reordering as described in the Automatic CICADA section. A lower mean and maximum percentile rank suggests that fewer ICs would need to be manually examined when using Manual CICADA as compared to standard manual IC classification. Therefore, lower means and maximum percentile ranks suggest a greater efficiency gain of manual classification through Manual CICADA.

#### 2.8.3 Quality control—benchmarks

The quality control benchmarks included subject-level QC (i.e., mFD for each subject) to functional connectivity (QC-FC) correlations, QC distance dependence correlations, and noise profile correlations (Table 1). Modularity (Q), mFD to modularity correlation (QC-Q), and loss of temporal degrees of freedom (tDOF) are also supplied in the Supplementary Material (Supplementary Tables 2 and 3) given their common inclusion in other literature comparing fMRI denoising success (Ciric et al., 2017; Golestani & Chen, 2024; Zhukovsky et al., 2022). Functional connectivity was assessed using regions of interest (ROIs) from the Brainnetome atlas (Fan et al., 2016). Only ROIs that were fully contained within each subject’s functional mask were included. Functional connectivity was calculated as the correlation between the average timeseries in each pair of ROIs. As described previously (see section 2.1.3), noise-induced connectivity is characterized by the strength of the correlation more than the sign. Therefore, both QC-FC correlations and QC distance dependence correlations are assessed by their mean magnitude values.

**Table 1.** Quality control metrics summary for evaluating automatic CICADA.

QC metric	Calculation	Improved denoising direction^a^	Motion-centric or non-motion-centric^b^
QC-FC^c^	$\bar{\left|r\left(FC,\;mFD\right)\right|}$	Lower	Motion-Centric
DD^c^	$\bar{\left|r\left(FC,\;Dist\right)\right|}$	Lower	Motion-Centric
Noise profile: region-based			
Edge^d^	$\bar{\left|r_{Edge}\right|}$	Lower	Motion-Centric
Outbrain^d^	$\bar{\left|r_{Outbrain}\right|}$	Lower	Non-Motion-Centric
Subependymal^d^	$\bar{\left|r_{Subependymal}\right|}$	Lower	Non-Motion-Centric
CSF^d^	$\bar{\left|r_{CSF}\right|}$	Lower	Non-Motion-Centric
Susceptibility^d^	$\bar{\left|r_{Susceptibility}\right|}$	Lower	Non-Motion-Centric
NotGM^d^	$\bar{\left|r_{NotGM}\right|}$	Lower	Both
Noise profile: confound-based			
FD-GM^d^	$\bar{\left|r\left(\left|GM^{\prime }\right|,\;FD\right)\right|}$	Lower	Motion-Centric
DVARS-GM^d^	$\bar{\left|r\left(\left|GM^{\prime }\right|,\;DVARS\right)\right|}$	Lower	Motion-Centric
DS^d^	$\frac{\bar{\left|r_{GM}\right|}/\bar{\left|r_{NotGM}\right|}}{\bar{\left|r_{GM\_O}\right|}/\bar{\left|r_{NotGM\_O}\right|}}$	Higher	Both

a Improved denoising direction refers to the direction of the given quality control metric that would indicate better denoising.

b Motion-centric refers to a metric that is heavily influenced by motion. Non-motion-centric metrics may still be influenced by motion but typically less so than the motion-centric metrics.

c The given metric is calculated per region-of-interest (ROI) pair across all participants. The final reported group metric is the mean ROI-pair metric.

d The given metric is calculated per subject. The final reported group metric is the mean subject metric.

Abbreviations: $\bar{\left|x\right|}$: the mean absolute value for given variable x; QC: Quality Control; QC-FC: Quality Control and functional connectivity correlation, mean magnitude; FC: Functional connectivity; mFD: median framewise displacement per subject; DD: Distance Dependence – FC and distance between ROI centroids, mean magnitude; Dist: Distance between ROI centroids; CSF: Cerebral Spinal Fluid; NotGM: not gray matter; FD-GM: framewise displacement to GM noise profile; DVARS-GM: temporal Derivative of root mean square VARiance over voxels to GM noise profile; DS: The denoising success parameter; O: “original data”.

##### 2.8.3.1 QC-FC correlations

QC-FC correlation helps assess the mitigation of head motion influence on functional connectivity estimates (Ciric et al., 2017). For each ROI pair, the QC-FC was calculated as the correlation between the subjects’ mFD and connectivity. A higher QC-FC magnitude indicates a greater association between motion and connectivity. Across all ROI pairs, the mean QC-FC magnitude for each method was also calculated. Overall, a significantly smaller mean QC-FC magnitude for a given method suggests greater removal of motion impact on connectivity.

##### 2.8.3.2 Distance dependence correlations

Distance dependence quantifies the preservation of intrinsic spatial connectivity patterns (Power et al., 2012; Satterthwaite et al., 2012). For each subject, distance dependence was calculated as the correlation between the ROI pairs’ connectivity and the Euclidean distance between their centroids. A higher distance dependence magnitude for a subject indicates a greater association between brain region distance and connectivity. Across all subjects, the mean distance dependence magnitude for each method was also calculated. Overall, a significantly smaller mean distance dependence magnitude for a given method suggests greater removal of distance impact on connectivity. While not directly related to motion, distance dependence, alongside QC-FC, is heavily influenced by motion (Power et al., 2015). Thus, both QC-FC and distance dependence are referred to as “motion-centric” metrics from hereon. Consequently, quality control metrics that are not as heavily influenced by motion are hereby referred to as “non-motion-centric.”

#### 2.8.4 QC—noise profile correlations

The eight noise profile correlation distributions calculated by Automatic CICADA were also compared across denoising methods. As previously described (section 2.1.3), these include six region-based noise profile correlations (edge, outbrain, subependymal, CSF, susceptibility, and notGM) and two confound-based noise profile correlations (FD-GM and DVARS-GM). Of note, motion artifact in IC analysis is often observed as a ring around the edge of the brain or as stripes close to the edge of the field of view (Griffanti et al., 2017). Therefore, alongside FD-GM and DVARS-GM noise profiles, as well as QC-FC and distance dependence, the edge region-based noise profile is also considered a motion-centric measurement. The other region-based noise profiles, then, are considered non-motion-centric measurements. It is also important to note, though, that motion effects are not limited to the edge of the brain (Maknojia et al., 2019; Power et al., 2012). Therefore, even the non-motion-centric noise profiles may commonly include varying degrees of motion artifact, albeit typically less so than the edge noise profile.

For each subject, a greater mean magnitude of the noise profile correlations suggests greater identifiability of the given noise profile. Across all subjects, the mean noise profile correlation magnitudes were also calculated. Overall, a smaller mean noise profile correlation magnitude for a given method may suggest greater removal of the given noise profile.

Whereas retention of correlations outside of GM following denoising would primarily be driven by noise, retention of within-GM correlations could be due to retention of both noise and signal. If GM neural signal correlations are maintained following successful denoising, we would therefore expect that notGM correlation magnitudes would be reduced more than within-GM correlations. A denoising method that more successfully removes noise while retaining signal, therefore, would be expected to result in a greater ratio between GM and notGM profile correlation magnitudes. This ratio of the mean magnitude of GM profile correlations to the mean magnitude of notGM profile correlations can then be compared against the original image before denoising to quantify “denoising success” (DS). Therefore, a measure of denoising success for subject s, *DS_s_*, can be calculated as:

$$DS_{s}=\frac{\bar{\left|r_{GM}\right|}/\bar{\left|r_{NotGM}\right|}}{\bar{\left|r_{GM\_O}\right|}/\bar{\left|r_{NotGM\_O}\right|}}$$ (1)

where $\bar{\left|r_{GM}\right|}$ and $\bar{\left|r_{GM\_O}\right|}$ are the GM profile correlation mean magnitudes for the given method and the original data before denoising, respectively. Meanwhile, $\bar{\left|r_{NotGM}\right|}$ and $\bar{\left|r_{NotGM\_O}\right|}$ are the notGM profile correlation mean magnitudes for the given method and the original data before denoising, respectively. As such, *DS_s_* was calculated for each subject alongside the mean *DS* for each denoising method. The denoising methods’ *DS_s_* were then compared with a signed rank test. Overall, a significantly greater *DS* for a given method suggests improved signal-to-noise enhancement.

Of note, the rationale underlying the DS metric can also be considered through the lens of ICA-based denoising itself. Only ICs that demonstrate higher correlations within GM are retained as signal, while noise ICs, which may include correlations both within and outside of GM, are removed. Therefore, ICA denoising should typically increase the relative ratio of the mean magnitude of GM profile correlations to the mean magnitude of notGM profile correlations.

## 3 Results

IC classification accuracy of CICADA was compared to MIRC and the other ICA-based methods (FIX, ICA-AROMA) in the manual subsets. The QC benchmarks and noise profile correlation metrics of CICADA were compared against FIX, ICA-AROMA, and the original data before denoising. These metrics were evaluated in the high-motion rest, high-motion task, and low-motion rest datasets. Of note, while standard deviations are shown in most figures to depict the spread of results, this does not represent significance, as the significance testing was paired and non-parametric (see section 2.8). The demographics and motion characteristics for each dataset and manual subset are provided in Table 2.

**Table 2.** Demographic & motion characteristics for each dataset.

	High motion, visual task		High motion, resting state		Low motion, resting state	
	All	Manual subset	All	Manual subset	All	Manual subset
	M (SD)	M (SD)	M (SD)	M (SD)	M (SD)	M (SD)
Age (yrs)	44.1 (13.8)	41.7 (14.3)	44.1 (13.9)	41.0 (14.3)	24.4 (4.1)	23.8 (4.6)
Sex (F/M)	24/33	14/16	22/31	14/16	19/37	10/20
N	57	30	53	30	56	30
mFD (mm)	0.20 (0.11)	0.20 (0.11)	0.26 (0.15)	0.28 (0.16)	0.09 (0.05)	0.09 (0.04)

Abbreviations: M: mean; SD: standard deviation; yrs: years; F: female; M: male; N: number of subjects; mFD: group-level median framewise displacement.

### 3.1 IC classification accuracy

**Fig. 4.**
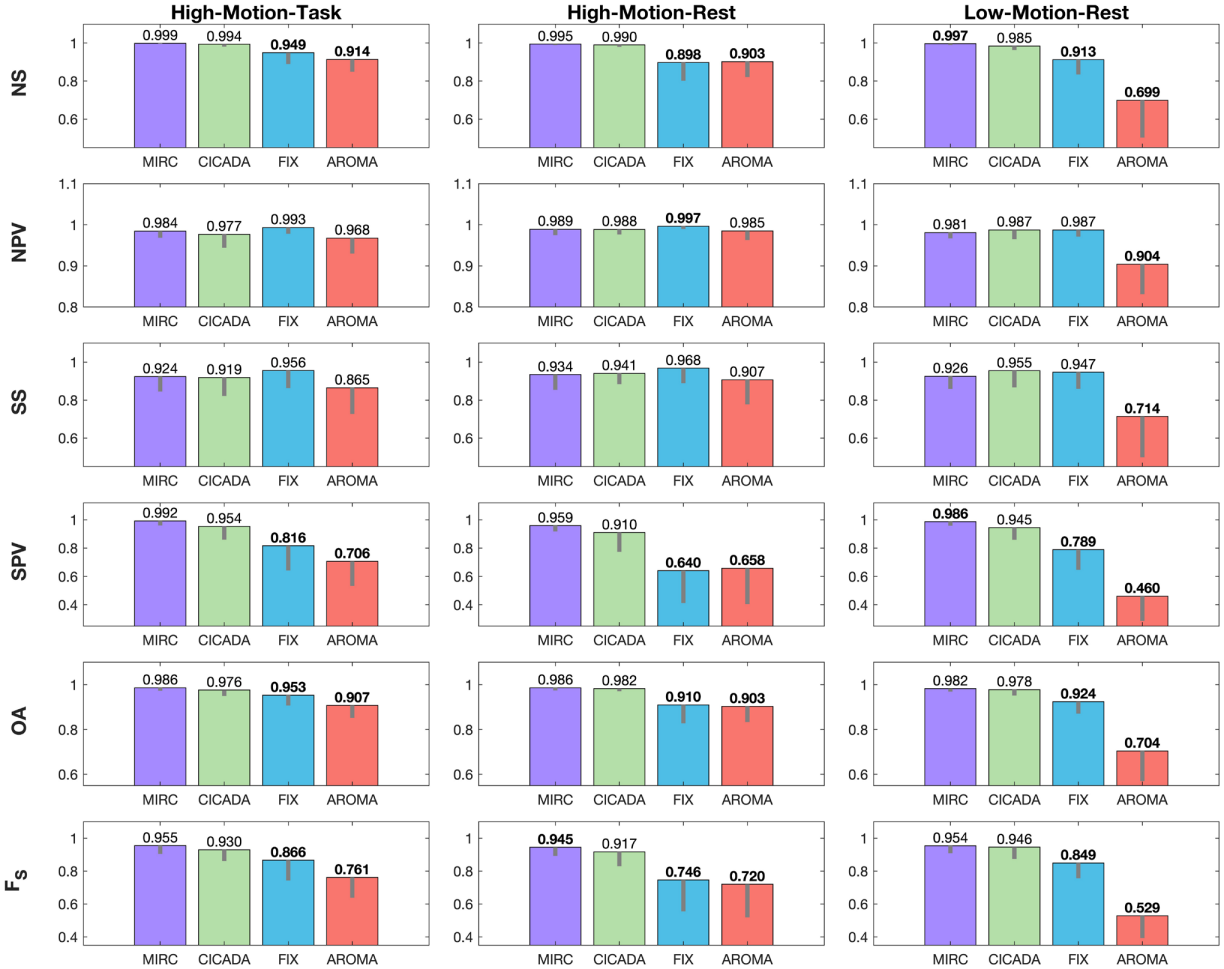
IC Classification Accuracy. Across nearly all accuracy measures, CICADA outperformed (values closer to one are better) both FIX and ICA-AROMA and performed similarly to manual individual rater classification (MIRC). All reported values are the mean subject accuracies. Bolded values denote significant differences between CICADA and each other method, Bonferroni corrected for the three comparisons (p < 0.017). The gray lines depict one standard deviation below the mean. Abbreviations: NS: noise sensitivity; NPV: noise predictive value; SS: signal sensitivity; SPV: signal predictive value; OA: overall accuracy; F_S_: signal F1 score.

CICADA was highly accurate in IC classification, performing similarly to manual individual rater classification and often outperforming both FIX and ICA-AROMA across all three datasets (Fig. 4; Supplementary Table 1). CICADA demonstrated comparable noise sensitivity to manual classification in both high motion datasets and greater noise sensitivity than FIX and ICA-AROMA in all three datasets (p < 0.001 in all cases). All methods showed similar noise predictive values to CICADA, but CICADA had lower noise predictive value than FIX in the high-motion rest dataset (p = 0.002) and greater noise predictive value than ICA-AROMA in the low-motion rest dataset (p < 0.001). Signal sensitivity was comparable to CICADA for all methods in both high-motion datasets, and only ICA-AROMA had lower signal sensitivity than CICADA in the low-motion rest data (p < 0.001). CICADA had similar signal predictive value to manual classification in both high-motion datasets and greater signal predictive value than FIX and ICA-AROMA in all three datasets (p < 0.001 in all cases). Importantly, in measures of overall IC classification success (i.e., OA, F_S_), CICADA performed better than FIX and ICA-AROMA across all datasets and was only outperformed by MIRC in the high-motion rest dataset (F_S_: p < 0.014). Altogether, Automatic CICADA was often more accurate than FIX and ICA-AROMA across high- and low-motion datasets, with accuracy approaching that of an individual manual rater.

### 3.2 Manual IC classification efficiency

Automatic CICADA decreased the number of ICs that would need to be examined to perform manual ICA denoising. Following the ranked ordering of ICs by NSP (see section 2.1.2), all ICs reflecting signal were contained, on average, in the first 14.5 out of 77 ICs. Furthermore, there were a maximum of four sequential noise ICs before the final signal IC. Therefore, in the current data, examining ICs in NSP rank order until 5 noise ICs in a row are encountered would be sufficient to accurately perform manual ICA denoising. Altogether then, Manual CICADA would result in a 75% reduction in the total number of ICs needed to be examined to perform manual ICA denoising for these datasets.

### 3.3 QC benchmarks

**Fig. 5.**
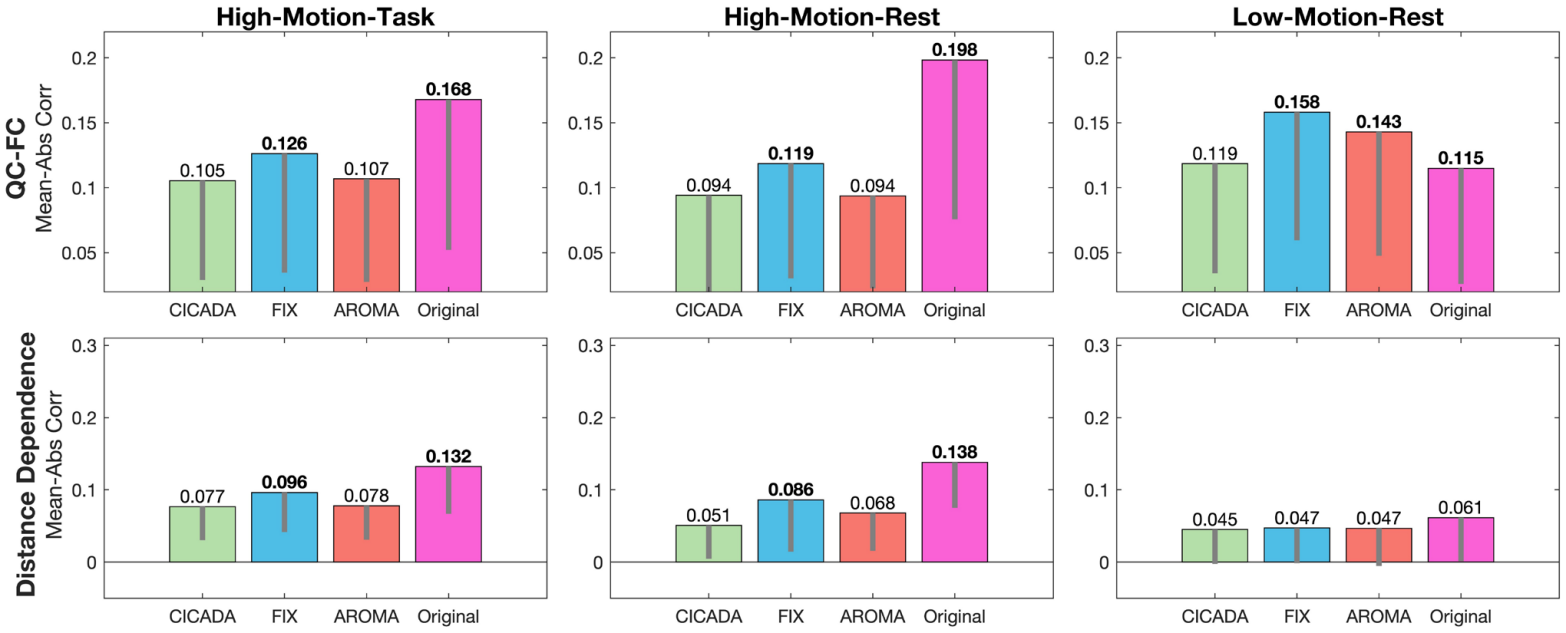
QC Benchmarks. Altogether, CICADA outperformed FIX and the original data, with ICA-AROMA performing the closest in comparison. For QC-FC and Distance Dependence, lower mean scores are typically better. Bolded values denote significant differences between CICADA and each other method, Bonferroni corrected for the three comparisons (p < 0.017). The gray lines depict one standard deviation below the mean. Abbreviations: QC-FC: Quality control (median FD) and functional connectivity mean correlation magnitudes; Distance Dependence: Functional connectivity of region-of-interest pairs and distance mean correlation magnitudes; Mean-Abs Corr: mean of the magnitude (absolute value) of the correlations; Abs Corr: magnitude of the correlation.

**Fig. 6.**
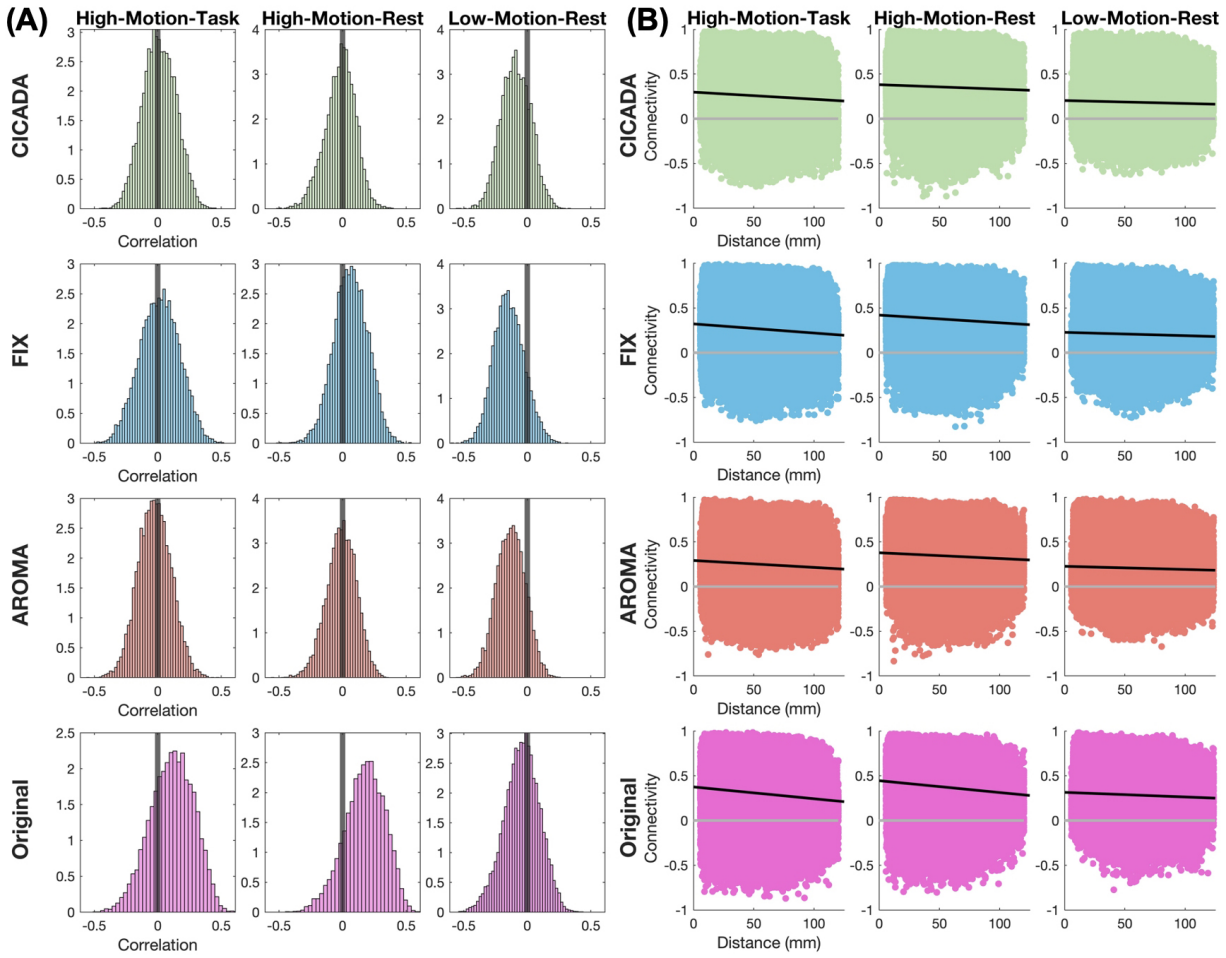
QC-FC and Distance Dependence Plots. (A) Histograms of median FD and functional connectivity correlations for each ROI pair for each method for each dataset with probability density function normalization. Better denoised data will have stronger correlations (whether positive or negative) removed and thus are characterized by narrower distributions that are typically centered near zero. A zero line is provided for reference. (B) Distance Dependence plots demonstrating the correlation (black line) between the distance between ROI pairs and function connectivity. Better denoised data typically has a smaller correlation. A zero line (gray line) is provided for reference.

Automatic CICADA resulted in improved QC benchmarks across the high-motion datasets, with results overall similar to ICA-AROMA, and often better than FIX (Fig. 5; Supplementary Table 2). First, CICADA reduced the mean QC-FC correlation magnitude (Figs. 5 & 6A; Supplementary Table 2) more than FIX across all datasets (p < 0.001), and more than ICA-AROMA in the low-motion rest dataset (p < 0.001). Of note, the original data had a lower mean QC-FC correlation magnitude than all the denoising methods for the low-motion rest data. For all the denoising methods in the low-motion rest dataset, however, the QC-FC score was negative (Fig. 6A). This suggests that motion was, perhaps appropriately, negatively associated with measured functional connectivity after denoising in the low-motion dataset. Second, CICADA reduced the distance dependence correlation magnitudes (Figs. 5 & 6B; Supplementary Table 2) more than FIX and the original data for both high-motion datasets (p’s < 0.001). CICADA and ICA-AROMA, meanwhile, showed similar performance in reducing distance dependence artifacts across all datasets. Altogether, in improving the QC benchmarks, CICADA was as successful as ICA-AROMA and more successful than FIX, especially in high-motion data.

### 3.4 QC—noise profile correlations

**Fig. 7.**
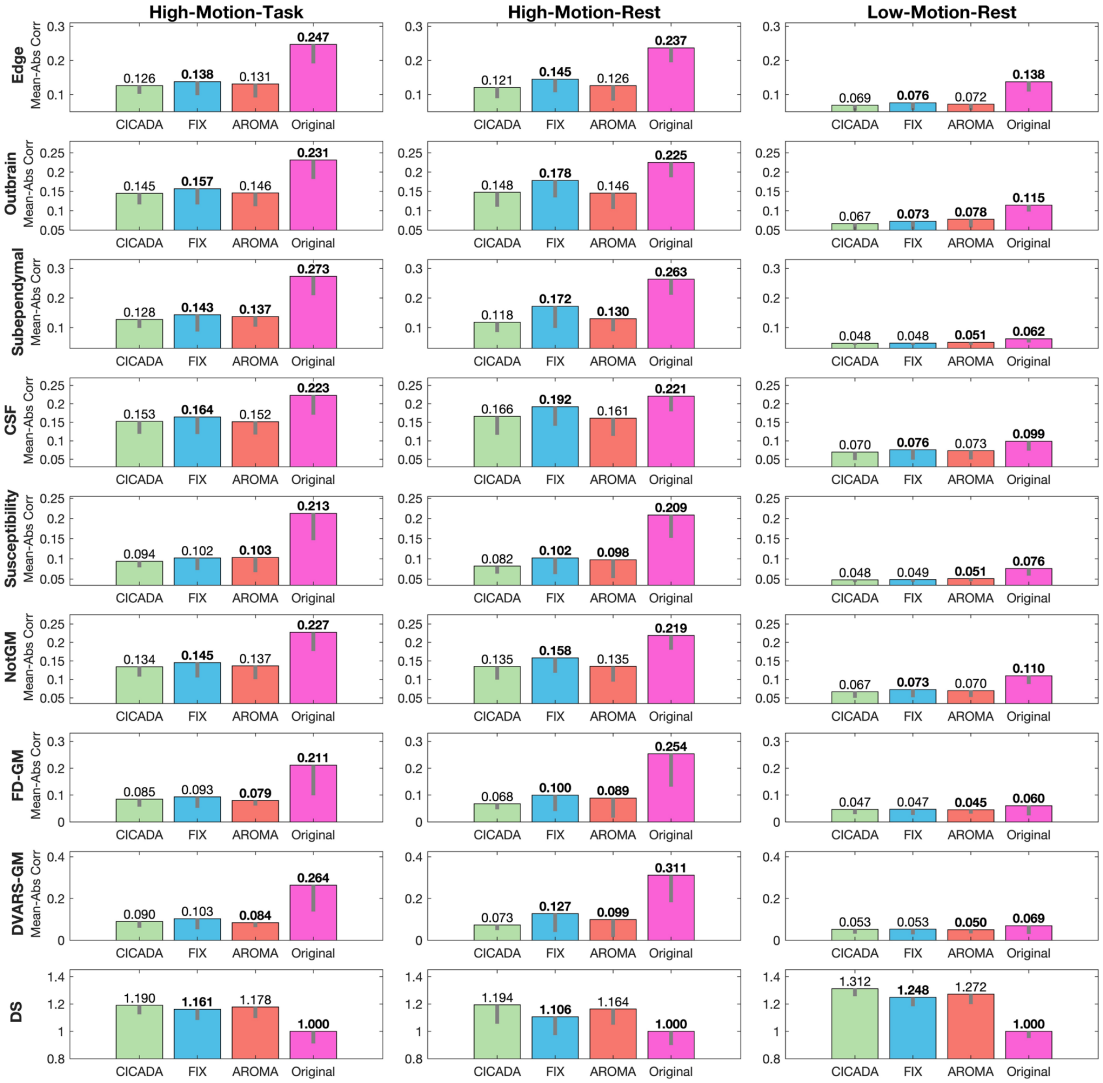
Noise Profile Correlations and Denoising Success. Overall, CICADA performed the best across all datasets (a lower value is better for all measures except DS where a higher value is better) and noise profiles and resulted in the highest denoising success. ICA-AROMA performed the most similarly to CICADA overall, especially in the high-motion datasets. FIX also performed similarly to CICADA in the low-motion-rest dataset. Bolded values denote significant differences between CICADA and each other method, Bonferroni corrected for the three comparisons (p < 0.017). The gray lines depict one standard deviation below the mean. Abbreviations: Mean-Abs Corr: mean magnitude of the correlations; FD-GM: framewise displacement to GM noise profile; DVARS-GM: temporal Derivative of root mean square VARiance over voxels to GM noise profile; CSF: cerebral spinal fluid; NotGM: not gray matter; DS: denoising success.

Compared to the other denoising methods, CICADA often resulted in similar, or better (lower), mean noise profile correlation magnitudes across most measures (Fig. 7). As previously described (section 2.1.3), region-based noise profiles were examined in image areas that encompass common sources of noise (edge, outbrain, subependymal, CSF, susceptibility artifacts, notGM). Confound-based noise profiles (FD-GM, DVARS-GM), meanwhile, were used to examine the association between GM voxel timeseries and motion-summary confounds (i.e., FD, DVARS). Compared to the original data, CICADA significantly reduced all region- and confound-based noise profile correlation magnitudes across all three datasets (Fig. 7; p < 0.001 in all cases).

Differences in noise profile performance between the three ICA-based methods were more variable. In the high-motion rest data, CICADA was significantly better than FIX in improving noise profile correlation magnitudes across all metrics (p < 0.001 in all cases). In the high-motion task and low-motion rest datasets, CICADA outperformed FIX in reducing the noise profile correlation magnitudes for the edge, outbrain, CSF, and notGM regions (p < 0.001 in all cases). CICADA and ICA-AROMA, meanwhile, demonstrated similar performance in reducing region- and confound-based noise profile correlations, with only slight differences across all mean correlation magnitudes. CICADA outperformed ICA-AROMA in reducing noise profile correlations for the subependymal (high-motion task: p = 0.005; high-motion rest: p < 0.001; low-motion rest: p < 0.001) and susceptibility (high-motion task: p = 0.009; high-motion rest: p = 0.001; low-motion rest: p < 0.001) regions in all three datasets. ICA-AROMA, meanwhile, was more successful than CICADA in reducing some noise profile correlation magnitudes for the high-motion task (FD-GM, p = 0.007; DVARS-GM, p = 0.002) and low-motion rest (FD-GM, p = 0.009; DVARS-GM, p = 0.008) datasets, though the maximum mean magnitude correlation difference was only 0.006 (Fig. 7; Supplementary Table 4). In contrast, CICADA was more successful than ICA-AROMA in reducing similar noise profile correlations for the high-motion rest (FD-GM, p = 0.013; DVARS-GM, p = 0.016) datasets, though the maximum mean magnitude correlation difference was only 0.016 (Fig. 7; Supplementary Table 4). In the low-motion dataset, CICADA was also more successful than ICA-AROMA in reducing outbrain noise profile correlations (p < 0.001). Finally, the summary measure of denoising success, DS, successfully captured the patterns indicated by the individual noise profiles. Across all datasets, CICADA showed greater DS than FIX and the original data, but similar DS to ICA-AROMA.

## 4 Discussion

The current work introduces CICADA as a novel, comprehensive, and robust tool to automate the challenging manual ICA denoising method. Across the three datasets, CICADA overall classified ICs more accurately and denoised the data more comprehensively than the most widely used semi-automated (FIX) and automated (ICA-AROMA) ICA-based denoising methods. Furthermore, the Manual CICADA pipeline greatly decreased the time required for manual IC classification. Altogether, this demonstrates that CICADA is an efficient and accurate method for denoising fMRI data.

Automatic CICADA denoising closely matched manual denoising. CICADA achieved 97.9% mean IC classification accuracy, similar to manual rater classification (MIRC mean IC accuracy = 98.5%), across all datasets. CICADA was also nearly as precise and sensitive in retaining neural signal-dominant ICs as MIRC across all datasets, as assessed by the F_S_ score. Across QC benchmarks, noise profile correlations, and denoising success, CICADA performed nearly equivalent to manual denoising (see Supplementary Material section 3.1). Finally, the Manual CICADA pipeline expedited manual ICA denoising implementation by greatly reducing the number of ICs requiring examination. Altogether, these results suggest that CICADA can effectively automate manual denoising, replicating its noise removal and signal retention.

CICADA outperformed the other ICA-based methods in IC classification overall accuracy and F_S_ scores. Specifically, CICADA was more precise with, and not less sensitive to, classifying neural signal-dominant ICs compared to both FIX and ICA-AROMA. For FIX, the lower F_S_ was primarily due to decreased precision (signal predictive value), suggesting FIX retained more noise despite similar sensitivity to CICADA. This increased noise retention likely stems from the limited size of the manually classified training dataset (N = 10 per dataset). Enlarging the training dataset would likely improve FIX’s precision but would also increase time-intensiveness of the semi-automated method. Meanwhile, for ICA-AROMA, the lower F_S_ score was due to both decreased sensitivity and precision, thereby retaining more noise and less signal than CICADA. This decreased accuracy is likely due to ICA-AROMA targeting mainly motion-related noise. Therefore, noise-dominant ICs that are independent to motion are more likely to be retained, and neural-signal dominant ICs that are associated with motion may be removed. Of note, the overall variability in accuracy for CICADA, MIRC, and FIX did not appear to be substantially different between the high-motion and low-motion datasets (see gray lines in Fig. 4). In contrast, ICA-AROMA variability in IC classification accuracy appeared to be higher in the low-motion dataset (see gray lines in Fig. 4). Altogether, while all three ICA-based methods classified ICs well, CICADA’s performance was closer to that of manual individual raters than FIX or ICA-AROMA.

CICADA was also highly successful in reducing motion-related noise. CICADA outperformed FIX in most motion-centric measures (QC-FC, distance dependence edge, FD-GM, and DVARS-GM) across most datasets, except in the low-motion dataset, where FIX performed similarly to CICADA (e.g., FD-GM, DVARS-GM). This similarity likely reflects better generalizability of FIX’s manual training dataset in low-motion conditions, due to reduced subject-level variability. Meanwhile, compared to ICA-AROMA, one of the most effective methods for reducing motion effects (Pruim, Mennes, Buitelaar, et al., 2015), CICADA performed similarly in most motion-centric parameters across all datasets, with the main exception of QC-FC in the low motion dataset where CICADA outperformed ICA-AROMA. This improved motion removal by CICADA is likely due to the reduced subject motion, which diminishes motion effects across ICs and makes non-motion-related noise more dominant. As a result, CICADA may be more likely than ICA-AROMA to identify and remove these ICs and their associated noise, leading to more comprehensive motion effect reduction. Additionally, there were small differences in FD-GM and DVARS-GM between CICADA and ICA-AROMA in both high- and low-motion datasets (Fig. 7; Supplementary Table 4). Of note, QC-FC and Distance Dependence demonstrated high subject variability, with standard deviations often near or above the mean (see Figs. 5 and 6). While this variability may warrant caution in interpretation, these metrics may still serve as valuable group-level benchmarks for assessing motion-related noise removal. Overall, CICADA may reduce motion-related noise as well as ICA-AROMA and better than FIX.

CICADA was also effective at reducing non-motion-related noise. Compared to FIX, CICADA performed marginally better in reducing correlations with most non-motion-centric noise profiles (i.e., outbrain, subependymal, susceptibility, CSF, and notGM) across all datasets. These differences were most pronounced in the high-motion-rest dataset and least evident in the low-motion dataset. As with motion-related noise, this likely reflects higher inter-subject variability and lower generalizability of FIX’s training data in high-motion conditions. Meanwhile, compared to ICA-AROMA, CICADA also reduced non-motion-related noise marginally better, especially in the low-motion dataset. This is perhaps unsurprising, as motion will impact all ICs, including those with primarily non-motion-related noise. Therefore, since ICA-AROMA is sensitive to motion artifacts, it should remove more non-motion-centric ICs in high-motion data than low-motion data. Altogether, these findings suggest that CICADA can reduce non-motion-related noise better than FIX, especially in higher motion data, and better than ICA-AROMA, especially in lower motion data.

Finally, CICADA performed well in retaining neural signal, as assessed by F_S_ and the novel denoising success (DS) parameter. CICADA’s significantly higher F_S_ scores than both FIX and ICA-AROMA across all datasets indicates a better ratio of signal retention to noise removal. Furthermore, CICADA outperformed FIX in DS across all three datasets, and performed at least as well as ICA-AROMA. Overall, FIX was more likely to label noise ICs as signal in all three datasets (i.e., had lower signal predictive value). Meanwhile, ICA-AROMA’s reduced F_S_ scores reflect both reduced signal predictive value and sensitivity as compared to CICADA. This is particularly evident in the low motion dataset, where ICA-AROMA performed significantly worse than CICADA in both signal predictive value and sensitivity. Notably, however, while ICA-AROMA was both less sensitive and precise in detecting signal, it did not perform significantly worse than CICADA in the DS parameter after correcting for multiple comparisons (see Supplementary Material section 3). This may indicate that both the noise and signal ICs that ICA-AROMA misclassified tended to have relatively lower denoising importance (e.g., misclassified signal and noise ICs had relatively low signal variance impact or minimally significant brain spatial overlap). Overall, CICADA may improve neural signal identifiability better than FIX in both high-motion and low-motion datasets. Similarly, CICADA may perform as well as, or better than, ICA-AROMA in neural signal identifiability, especially in lower-motion datasets.

CICADA has several methodological strengths. CICADA offers high analytic flexibility through adjustable detrending, temporal filtering, and smoothing, as well as providing post-denoising quality control tools at the subject and group levels. Furthermore, CICADA provides an expedited method to fully implement manual IC classification by greatly reducing the number of ICs that need to be examined. Unlike FIX, CICADA is fully automated and does not require data training. Additionally, compared to ICA-AROMA, CICADA more comprehensively targets broad sources of fMRI noise beyond motion effects. Altogether, the methods presented in CICADA were designed to overcome limitations of current ICA-based denoising tools.

The current findings should be considered within the context of study limitations. CICADA has not been evaluated for other types of fMRI task designs or in clinical populations outside of those in the datasets presented here. Additionally, methods were optimized using an adult brain template and have not yet been adapted for pediatric populations. CICADA may be less accurate in IC classification for data with pathology affecting tissue segmentation and normalization (e.g., tumors or stroke). Although CICADA was not evaluated on a high-motion multiband-acquired dataset, its accuracy scores did not differ significantly when the multiband-acquired dataset was split into higher- and lower-motion subsets (see Supplementary Table 5); similarly, CICADA’s accuracy scores did not correlate significantly with motion in the multiband-acquired dataset (see Supplementary Table 6). CICADA performance was evaluated against manual IC classification of multiple raters which, while a practical reference, does not guarantee complete accuracy. Data were not pre-smoothed for all ICA-based methods to remain consistent in performance comparisons; this may disadvantage ICA-AROMA, however, as it was designed for pre-smoothed data. Additionally, regression of motion confounds in FIX (an optional step) was not applied to remain consistent in performance comparison, but implementing it might have improved FIX’s denoising performance. Finally, while FIX and ICA-AROMA are the most widely used automated ICA denoising methods, we did not compare CICADA to all available ICA-based denoising tools. Two alternatives that are less computationally intensive than CICADA include ALT (Zhukovsky et al., 2022) and SOCK (Bhaganagarapu et al., 2013). Similar to CICADA, both ALT and SOCK quantify IC spatial map and power spectrum qualities to automatically classify ICs. CICADA, however, uses more quantifications of the spatial maps, power spectra, and timeseries to more specifically follow manual IC classification guidelines and comprehensively identify noise sources. Furthermore, neither ALT or SOCK are currently as widely used as either FIX or ICA-AROMA. Despite these limitations, the present results still strongly support the usefulness of CICADA.

In conclusion, CICADA generally outperformed the most widely-used automated ICA denoising methods, performed similarly to manual IC classification, and demonstrated that it can greatly ease manual classification implementation. These findings were largely consistent across both resting-state and task-based paradigms, as well as in higher-motion and low-motion data. Overall, CICADA is a comprehensive, accurate, flexible, and robust denoising tool that effectively automates the challenging manual ICA denoising gold-standard across a variety of fMRI datasets.

## Supplementary Material

Supplementary Material

## Data Availability

CICADA is publicly available and can be found on GitHub (https://github.com/keithcdodd/CICADA) along with instructions for its installation and use (“CICADA User Guide”) and example code structure (“example CICADA flow”). The HCPEP data used in this current work are available at https://www.humanconnectome.org/study/human-connectome-project-for-early-psychosis. fMRIPrep, used to preprocess the data before CICADA, is available at https://fmriprep.org/en/stable/. Future versions of CICADA will be extended to Python to increase accessibility.
