# Supplementary material for: CICADA: An automated and flexible tool for comprehensive fMRI noise reduction
Source: Imaging Neurosci (Camb). 2025 Aug 20;3:IMAG.a.114. doi: 10.1162/IMAG.a.114 (PMC12368612; doi:10.1162/IMAG.a.114)
Supplement: Supplementary Material [file IMAG.a.114_supp.pdf]

SUPPLEMENTARY MATERIAL

1. CICADA Application Details

The Comprehensive Independent Component Analysis Denoising Assistant (CICADA) is a novel ICA-based denoising method applicable to both resting-state and task-based fMRI data. CICADA is designed for use after preprocessing (including normalization to MNI space but without smoothing) but before statistical analyses. CICADA uses manual IC classification guidelines (Griffanti et al., 2017) (the gold-standard of ICA denoising) to help identify all commonly established sources of fMRI noise. Specifically, CICADA uses the IC spatial maps, timeseries, and power spectra to classify ICs as signal or various types of noise. CICADA can be split into three main sections (see main Figure 1 for an overview of sections). First is Automatic CICADA, which performs subject-level ICA denoising and quality control automatically. Second is Manual CICADA, an optional step, which allows users to perform a highly efficient version of manual IC denoising with quality control analyses. Third is Group CICADA, which performs group-level processing and quality control of the CICADA-denoised data. Altogether, CICADA aims to effectively automate the manual denoising gold standard, make the gold standard more efficient and approachable, and significantly ease quality control analyses following denoising.

1.1 CICADA Use Cases

CICADA is currently only compatible with data warped to the MNI 2009c asym adult template (Fonov et al., 2009). Therefore, it is not currently compatible with young pediatric data or data that cannot be warped to MNI 2009c asym space. CICADA works well with fMRIPrep preprocessing, but fMRIPrep is not required. CICADA is compatible with both resting-state and task data.

1.2 CICADA Installation

The most recent version of CICADA and instructions for use are available from the author’s Github page (https://github.com/keithcdodd/CICADA). Version v1.0.0 was the version used for the manuscript. Further installation details, as well as instructions on how to run CICADA, can be found there.

1.3 Automatic CICADA Methods

Automatic CICADA can be executed through either the Auto_CICADA or fmriprep_auto_CICADA functions, held within the “wrappers” directory in CICADA. On the GitHub, example scripts to call these functions appropriately for datasets is provided (example_CICADA_flow/example_code). In its simplest form, the only necessary inputs for Automatic CICADA is an output directory (where the results will be stored), the previously preprocess (but not yet denoised) functional image file, a functional image brain mask file, and a confounds file (see CICADA User Guide on the GitHub for more details). Ideally, the following anatomical information is also included: an anatomical file (e.g., T1), anatomical brain mask, and gray matter, white matter, and cerebral spinal fluid probability maps. If these anatomical images are not included, CICADA will automatically use MNI nonlinear asymmetric 09c image files. As such, if the data is not

in MNI nonlinear asymmetric 09c space, then the anatomical brain mask, and gray matter, white matter, and cerebral spinal fluid probability maps are required (although the anatomical file is not required and does not have to be a T1). If the image files are from task-based data, CICADA may also benefit from, but does not require, inputting a task events file. The task events file for CICADA is of the same format as in BIDS file formatting. In short, it must be a .tsv, and for CICADA, must at least include the following columns: onset, duration, and trial_type. Of note, baseline conditions should be labeled specifically as “baseline” in the trial_type column. This allows CICADA to ignore baseline when modeling hemodynamic response functions. To accommodate random event-related task designs, a different task events file can be provided for each fMRI scan. If the task design includes impulse functions instead of blocks, the duration can be set as either 0 or the TR length in seconds. An example file is shown below in Supplementary Figure 1.

onset	duration	trial_type
0	16	baseline
32	16	task_1
96	16	task_2
16	16	task_3
48	16	baseline
128	16	task_1
112	16	task_2
80	16	task_3
64	16	baseline

Supplementary Figure 1. *Task Events File Formatting Example.*

Automatic CICADA methods are all contained within three main basescripts. The methods are described in detail below. The general flow of the Automatic CICADA Pipeline is also displayed in Supplementary Figure 2.

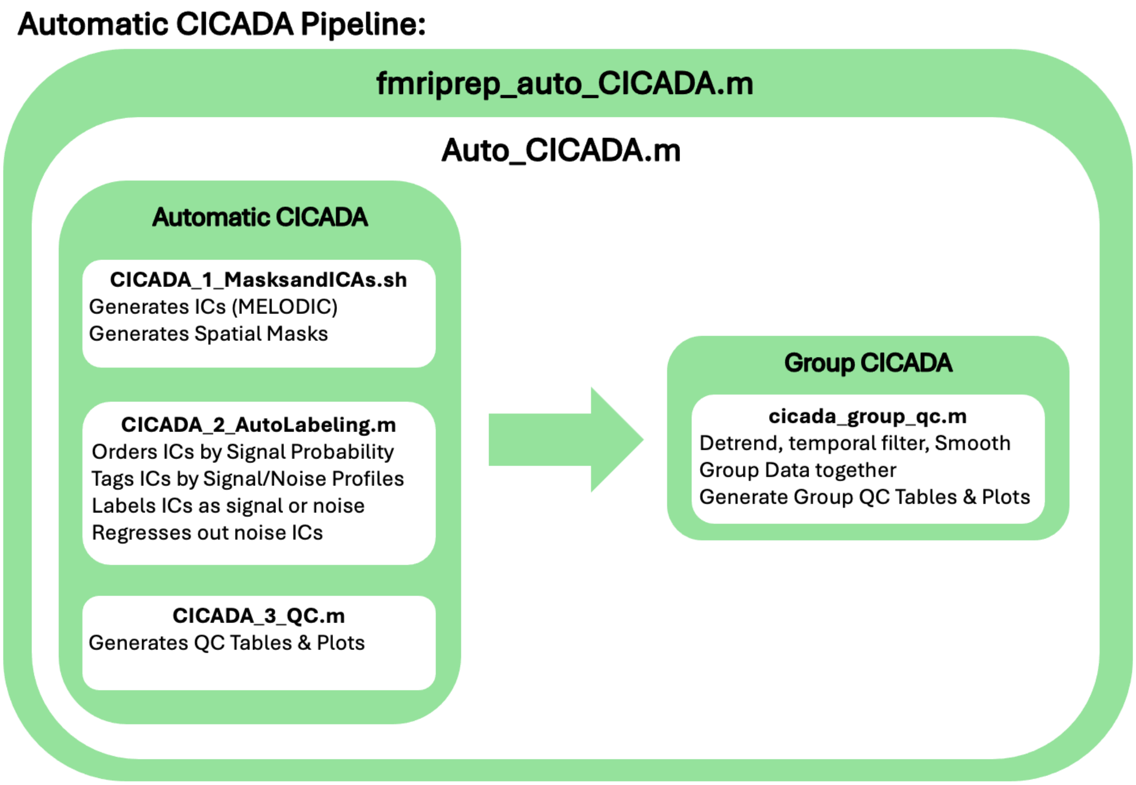

Supplementary Figure 2. *Script Flow for Automatic CICADA Pipeline.* Of note, Auto_CICADA.m is sufficient to run the full Automatic CICADA pipeline. The fmriprep_auto_CICADA.m may just offer an easier method to implement the Automatic CICADA pipeline for datasets that have been preprocessed with fmriprep.

1.3.1 Basescript 1: CICADA_1_MasksandICAs.sh

Broadly, the first basescript creates anatomical and functional masks, performs FSL's MELODIC to generate ICs, and provides relevant calculations of these ICs, using the previously created masks, to prepare for IC classification in the second basescript.

Many new masks are generated by the first basescript. First, a new functional mask is created to ensure that areas outside of the brain tissue (but generally excluding the skull) are included. This helps ensure that CICADA will be able to characterize both cerebral sinus flow and edge artifact. This is accomplished by first taking the union of the original functional mask and the anatomy mask (resampled to functional space). Second, the output from the first step is masked both by a 6 mm gaussian smoothed anatomical mask and a lightly thresholded (all voxel values below the 5th percentile removed) unmasked functional volume to form the final functional mask. Altogether, this creates a functional mask large enough to include sinus flow and edge artifact, but without retaining large amounts of other material (e.g., skull, eyes, etc.). The unmasked functional volume is then masked by this new functional mask (which is used as the functional mask for all future calculations as well).

Several region-based probability maps and masks are then created. In each case, a probability volume (an approximation of the relative chance that a voxel belongs to the given region) is created. Then, a mask of this region is generated by thresholding at the

robust 67th percentile of voxel intensities using *fslmaths -thrP*. The first region-based probability map generated in this way is the susceptibility probability map. In short, voxels from the functional volume with intensities at or above the robust 67th percentile are assigned a susceptibility probability of 0%. Susceptibility probabilities then increase up to 100% as the voxel values decrease to the 0th percentile. The next generated region map is the functional volume edge probability map. To create the edge probability map, both the susceptibility mask and an eroded and smoothed functional mask are removed from the functional mask. New region maps for gray matter (GM), white matter (WM), and cerebral spinal fluid (CSF) are next calculated. The original corresponding tissue probability maps (provided by the user, often previously calculated from the T1 anatomical) are first resampled, then the edge and susceptibility probabilities are subtracted and then masked by the functional mask. Of note, while the original tissue probability maps can be provided by the user to CICADA, CICADA will default to using either the tissue probability outputs from fMRIPrep (if they exist) or the MNI 2009c tissue probability maps (Fonov et al., 2009) (included with CICADA). Following generation of the new GM, WM, and CSF region maps, a subependymal probability map is calculated. In short, CSF and WM are masked by an eroded anatomy mask. The GM probability is then subtracted out and then smoothing is applied. The overlap between these modified CSF and WM probability volumes are then used to estimate the subependymal region. Next, a notGM region is calculated by subtracting the GM probability map from the functional mask. An “inbrain” region is generated by subtracting the subependymal probability map from a combined GM and WM region. An “outbrain” region is then formed from subtracting this “inbrain” probability map from the functional mask. Finally, an “outbrain_only” probability map (a region containing mostly outside cerebral sinuses and not the inner CSF) is created by subtracting the CSF, subependymal, edge, and susceptibility probability maps, as well as removing an eroded anatomy mask, from the outbrain probability map. This concludes the major region/mask generations of the first basescript. See Figure 2 in the main manuscript for an example of the output volume from this basescript.

After creating the relevant masks, the first basescript then performs FSLs MELODIC (Beckmann & Smith, 2004) to generate the ICs. Following MELODIC, voxelwise spatial map probabilities were obtained directly from MELODIC’s output. For potential visual assessment by the user, the individual 3D spatial probability maps for each independent component were optionally merged into a single 4D file. A thresholded version was also generated, retaining only voxels with a probability of 99% or higher of belonging to a given IC. Neither the merged nor the thresholded maps were used in subsequent analyses.

Estimates of IC smoothness were then computed. Two different IC spatial maps are generated to later calculate smoothness. First, a non-thresholded IC z-stat map is created from MELODIC outputs and absolute valued. Second, the non-thresholded IC z-stat map is smoothed (6 mm FWHM Gaussian kernel) and absolute valued. The second basescript later uses these two maps to calculate a smoothness parameter by dividing the summation of the non-thresholded IC z-stat map by the smoothed one. In short, the smoother the IC spatial map data is, the less data is “lost” to 6 mm FWHM Gaussian smoothing (e.g., positive signal is “less cancelled out” by negative signal).

The first basescript then also resamples a brain network template (Guzmán-Vélez et al., 2022) to the functional space.

The final part of basescript one involves calculating the IC spatial map overlap with each relevant mask generated near the start of this script (alongside the networks from the adult brain network template, if desired). Spatial map overlap is calculated by multiplying the magnitude of each melodic IC of each voxel (i.e., non-thresholded and absolute-valued) by each relevant region mask. The FSL command *fslstats* then outputs the mean of the non-zero voxels, as well as the number of non-zero voxels. This then sets up the inputs for basescript two, which will calculate the total overlap (mean of non-zero voxels multiplied by number of non-zero voxels) for each relevant region from these values.

1.3.2 Basescript 2: CICADA_2_AutoLabeling.m

Broadly, the second basescript classifies the ICs and applies nonaggressive denoising. First, a smaller, more constrained, functional mask is generated. The purpose of this constrained mask is to help later generate an improved group functional mask. Next, an HRF response is estimated using a double gamma HRF. If the functional volume is a task-based scan and task events are provided, HRF response estimates for each trial-type and across all trials are also generated through convolution with the estimated HRF. Next, the second basescript uses the outputs of the first basescript to calculate the general IC spatial overlap with each relevant region and network. The relative proportion of each region overlap compared to the total spatial IC map is also calculated. Smoothness ("smoothing retention") is estimated by dividing the summation of the non-thresholded IC z-stat map by the smoothed one (see "Basescript 1: CICADA_1_MasksandICAs.sh" section). Next, general power frequency proportions are calculated and categorized as low frequency (<0.008 Hz), BOLD (0.008-0.15 Hz), and high frequency (>0.15 Hz). The overlap between the IC power frequencies and the estimated HRF response power frequencies is also calculated ("HRF power frequency overlap"). Next, timeseries correlations to derivative of root mean square variance over voxels (DVARS), Framewise Displacement (FD, the version proposed by Power et al., 2012 (Power et al., 2012)), the six motion parameters, white matter, CSF, and global signal, are all calculated. These parameters will default to the estimations provided by fMRIPrep but can be input by the user if needed. Specifically, a detrended DVARS and FD are correlated to a detrended, differentiated, and absolute valued timeseries. This method is chosen as DVARS and FD are, by definition, differentiated and absolute valued parameters as well. A general tendency towards spikes in the IC timeseries is also estimated by the maximum absolute value of the normalized timeseries.

The second basescript then uses k-means clustering with three groups, to cycle through relevant noise profiles (see main manuscript for more detail on noise profiles), brain networks, and other related variables detailed above. The initial k-means starting points are given as the minimum, median, and maximum values. Thus, the ICs are clustered as either "high k-means", "medium k-means", or "low k-means" in that noise profile, network, or other variable. From here on, any mention of "k-means classified as high" or "k-means classified as low" refers specifically to if the ICs were clustered by k-means classification into the "high" or "low" groups, respectively. Many of these classifications

are also labeled as either “good” (highly likely to indicate neural signal) or “bad” (highly likely to indicate noise). In general, a “good k-means classification” for an IC refers to either “k-means classification as high” for a neural signal-associated measurement (e.g., Gray Matter spatial overlap) or “k-means classification as low” for a noise-associated measurement (e.g., CSF spatial overlap). Similarly, a “bad k-means classification” for an IC refers to either “k-means classification as low” for a neural signal-associated measurement or “k-means classification as high” for a noise-associated measurement. Basescript two then re-sorts the ICs, from high to low, based on the following equation for the relative Neural Signal Probability (NSP) as:

$$NSP = norm(S) * norm(GMO)^2 * norm(PSO)$$

where S is “Smoothness”, GMO is “Gray Matter spatial map Overlap”, and PSO is “Power Spectrum Overlap.” In each case, “norm” refers to a normalization (min-max scaling) of each parameter to values ranging from [0,1]. Overall, this equation takes advantage of the fact that neural signal is characterized by ICs with higher smoothness, higher gray matter overlap, and higher power frequency overlap with the estimated HRF response. GMO is squared to greater weigh its value, as gray matter overlap is likely more specific of neural signal than either smoothness or HRF power overlap. This can be inferred, for example, by the fact that certain noise profiles (e.g., Subependymal) can be highly smooth and have great HRF power overlap but not have high GM overlap.

After re-sorting the ICs from high to low based on NSP, CICADA loops through each IC to classify each IC as either (neural) signal, or noise. ICs were classified as signal if all of the following criteria are satisfied:

1. The IC is k-means classified as high in either GMO, PSO, or S.
2. The IC is either k-means classified as high in GMO or is not k-means classified high for any region-based noise profile.
3. The IC either has no noise-like k-means labels or is k-means classified as high in both GMO and either PSO or S.

Importantly, by following these three criteria, Automatic CICADA uses all calculated IC evaluations (not only GMO, PSO, S) to decide on signal or noise IC classifications. Furthermore, by examining the ICs in NSP order, Automatic CICADA can take advantage of a “tolerance value” to avoid needing to examine every ICs. Initially, the tolerance value is set to five by default. Whenever CICADA labels an IC as noise, the tolerance value is reduced by one. Similarly, when CICADA labels an IC as signal, the tolerance value is increased by one (but is never raised above the starting value). If the tolerance value reaches 0, CICADA stops looping through the ICs and labels the rest of the ICs as noise. CICADA will also not loop through ICs whose NSP is less than the mean IC NSP. Therefore, any IC whose NSP is less than the mean NSP will also be labeled as noise.

If less than two ICs are labeled as signal initially, CICADA will correct this by labeling the highest two NSPs as signal. This acts as a failsafe to the code structure. Later, Group CICADA (see “Group CICADA” section 2.3 in the manuscript) will also label each image that has less than 3 ICs labeled as signal as outliers to not be used.

Next, the second basescript creates a csv file (“IC_auto_checker.csv”) that details the selection process. The “IC_auto_checker.csv” includes the IC number, how CICADA labeled it (signal or noise), if the IC was high in signal or noise, any good (signal-like) or bad (noise-like) classifications given to the IC, and if the IC may have been grouped toward a particular brain network. An example image of part of one of these files is given here (Supplementary Figure 3). Of note, the “SignalLabel” column contains CICADA’s final decisions on IC classifications where “1” is signal, and “0” is noise. To perform Manual CICADA (see Manual CICADA Methods), the user would adjust the “SignalLabel” column and then resave the csv file as “IC_manual_checker.csv” before implementing the Manual CICADA scripts.

PotentialICs	SignalLabel	HighSignalLabel	HighNoiseLabel	Good_Tags	Bad_Tags	Networks_Tags
54	1	1	0	High_GM; High_Smoothing_Retention;	None	High_MedialVisual; High_De
25	1	1	0	High_GM; High_Smoothing_Retention;	None	High_DorsalAttention; High_
34	1	1	0	High_GM; High_Smoothing_Retention;	None	High_DefaultModeNetwork
26	1	1	0	High_GM; High_Smoothing_Retention;	None	High_FrontoParietal
55	1	1	0	High_GM; High_Smoothing_Retention;	None	High_DorsalAttention; High_
52	1	1	1	High_GM; High_Smoothing_Retention;	High_Spikiness	High_MedialVisual; High_Do
38	1	1	0	High_GM; High_Smoothing_Retention;	None	High_FrontoParietal
76	1	1	0	High_GM; High_Smoothing_Retention;	None	High_SensoryMotor; High_Ve
59	1	1	0	High_GM; High_Smoothing_Retention;	None	High_VentralAttention
43	1	1	0	High_GM; High_Smoothing_Retention;	None	None
42	1	1	0	High_GM; High_Smoothing_Retention;	None	High_FrontoParietal; High_D
60	1	1	0	High_GM; High_Smoothing_Retention;	None	High_SensoryMotor; High_Ve
39	1	1	1	High_GM; High_Smoothing_Retention;	High_Spikiness; High_DVARS_Corr	High_DefaultModeNetwork;
13	1	1	0	High_GM; High_Smoothing_Retention;	None	High_DefaultModeNetwork
31	1	0	1	High_GM; High_best_power_overlap_n	High_FD_Corr	High_DefaultModeNetwork
70	1	0	0	High_Smoothing_Retention; High_best	None	None
64	0	0	1	High_Smoothing_Retention; High_best	High_Spikiness	High_SensoryMotor
45	1	0	0	High_GM; High_Smoothing_Retention	None	High_VentralAttention
22	1	0	0	High_GM; High_Smoothing_Retention	None	High_MedialVisual; High_Do
73	1	1	0	High_GM; High_Smoothing_Retention;	None	High_DorsalAttention
36	1	0	0	High_GM	None	High_MedialVisual; High_Do
56	1	0	0	High_Smoothing_Retention; High_best	None	None
29	1	0	0	High_Smoothing_Retention; High_best	None	High_DorsalAttention
67	1	0	0	High_GM; High_Smoothing_Retention	None	High_VentralAttention
71	0	0	0	High_Smoothing_Retention	None	High_SensoryMotor
17	0	0	1	High_Smoothing_Retention; High_best	High_OutbrainOnly; High_Outbrain	High_FrontoParietal
50	1	0	0	High_Smoothing_Retention; High_best	None	None
23	0	0	0	High_Smoothing_Retention	None	High_SensoryMotor
69	0	0	0	None	None	High_DorsalAttention
18	0	0	1	High_Smoothing_Retention; High_best	High_OutbrainOnly; High_Outbrain	None
83	0	0	0	High_Smoothing_Retention	None	High_Subcortical
77	0	0	0	None	None	None
75	0	0	1	High_Smoothing_Retention; High_best	Low_GM; High_Subepe; High_CSF	None
37	0	0	1	None	Low_Smoothing_Retention	High_VentralAttention; High_
46	0	0	0	None	None	None
82	0	0	1	None	High_Subepe	None
68	0	0	1	None	High_Highfreq; Low_best_power_overl	None
9	0	0	1	None	High_Outbrain; High_DVARS_Corr; Hig	None

Supplementary Figure 3. *CICADA IC_auto_checker Example*

Finally, basescript two performs a few last actions to make it easier for users to examine the classification and output. CICADA creates structures to hold all relevant calculations, values, and classifications. CICADA also creates a “compare cleaning” table to compare the relative feature proportions of all ICs to just the ICs that CICADA labeled as signal. CICADA also generates a signal-to-noise ratio image and generates images containing all noise and all signal IC overlaps. Files to perform nonaggressive denoising (the standard default method) and aggressive denoising are saved and exported. Then, basescript two performs nonaggressive denoising via fsl_regfilt, alongside 8 parameter regression (six motion parameters + mean WM + mean CSF) and 9 parameter regression (8 parameter + mean global signal) for comparison. All relevant variables are saved and exported.

1.3.3 Basescript 3: CICADA_3_QC.m

Basescript three generates useful quality control (QC) analyses for each fMRI image. This assists in quality control analyses and in evaluating how well CICADA performed. This also helps inform a user if the IC selection for an image should be adjusted and rerun (“Manual CICADA”).

In creating QC plots, basescript three will default to comparing the CICADA-denoised file to the 8-parameter-denoised file, both generated by basescript two. The user, however, can provide the function with a different denoised file for comparison (e.g., 9 parameter denoising). First, basescript three records the number, and percentage of, ICs that were classified as signal. It then estimates the percent of variance retained in the data following CICADA denoising by summating the estimated signal variance (output by MELODIC) of each retained IC (classified as neural signal). Basescript three then calculates the relative noise profile correlations (e.g., correlations within the edge region, correlations within CSF, correlations between GM and framewise displacement). Finally, basescript three plots the noise profile correlations of both the CICADA denoised data and the comparison denoising data for reference (Supplementary Figure 4).

Other than QC plots, basescript 3 also outputs “network identifiability” NIfTIs. These files can also be found in the QC folder for the given subject. These images similarly compare CICADA denoised (“cleaned”), 8-parameter denoising (“compare”; regress 6 motion parameters, CSF, and WM), and the original data (“orig”) without any denoising. In short, these images visualize identifiability of 7 major networks (default mode, sensorimotor, visual, salience, dorsal attention, executive control, and limbic/reward). Identifiability is measured by correlating the mean signal of Brainnetome regions for each major network to the whole image (cleaned, compare, orig). From there, voxels are assigned a network label (i.e., 1-7) corresponding to the network with the highest correlation to each voxel. Voxels whose highest network correlation have a p-value > 0.5 (i.e., z-score < 0.67) are not assigned a network label. The network identifiability NIfTIs have three indices in the 4th dimension: the first is the cleaned (CICADA denoised) data, the second is the compare (8-parameter denoising), the third/last is the original data without any denoising. Overall, better denoised data will result in network connectivity that is easier to visually identify. An example visual is provided in Supplementary Figure 5.

The network seeds (“yeo_brainnetome_network_labels.nii.gz”) were generated by visually inspecting overlap between Brainnetome (Fan et al., 2016) regions and the Yeo 7-network template (Yeo et al., 2011). Brainnetome regions that demonstrated strong alignment with the given network in the Yeo atlas, and had strong support in the literature, were included. These network seeds are displayed at the top of Supplementary Figure 5, and they are included in CICADA. This concludes Automatic CICADA.

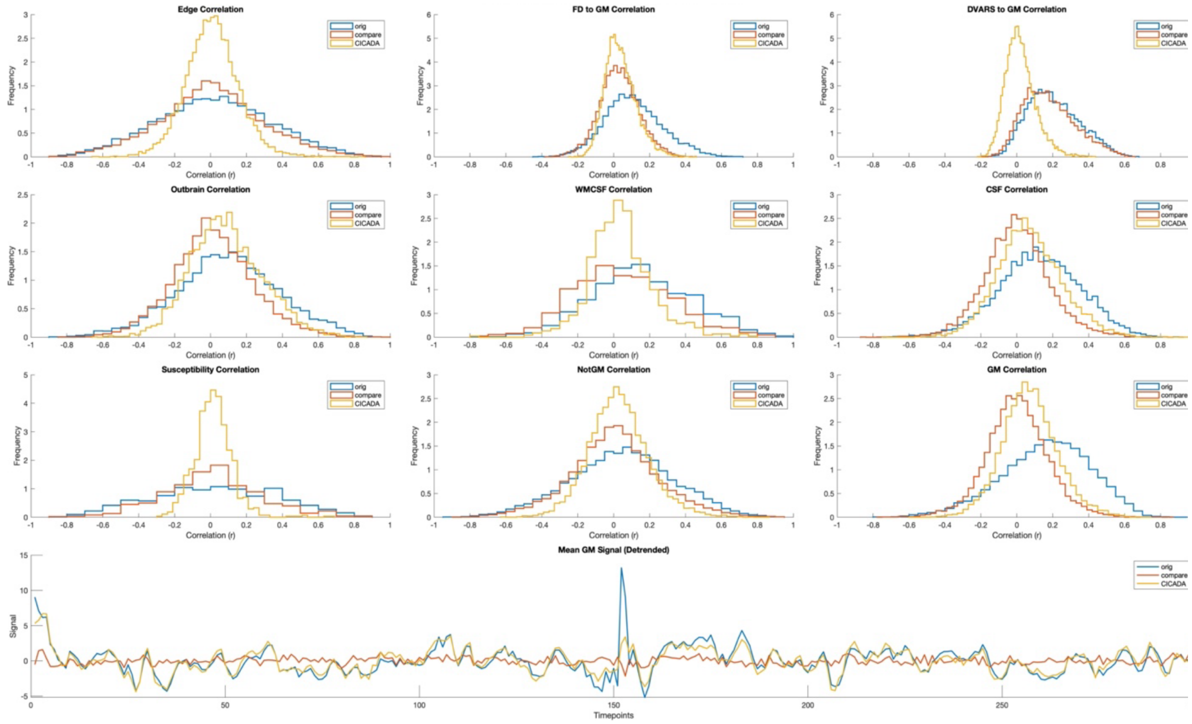

Supplementary Figure 4. *Noise Profile Correlation Histograms from Basescript 3.* These plots assist in QC analyses and can be used to compare CICADA to both the original data and other forms of denoising. In this example, CICADA is compared to 8p denoising as detailed in the manuscript.

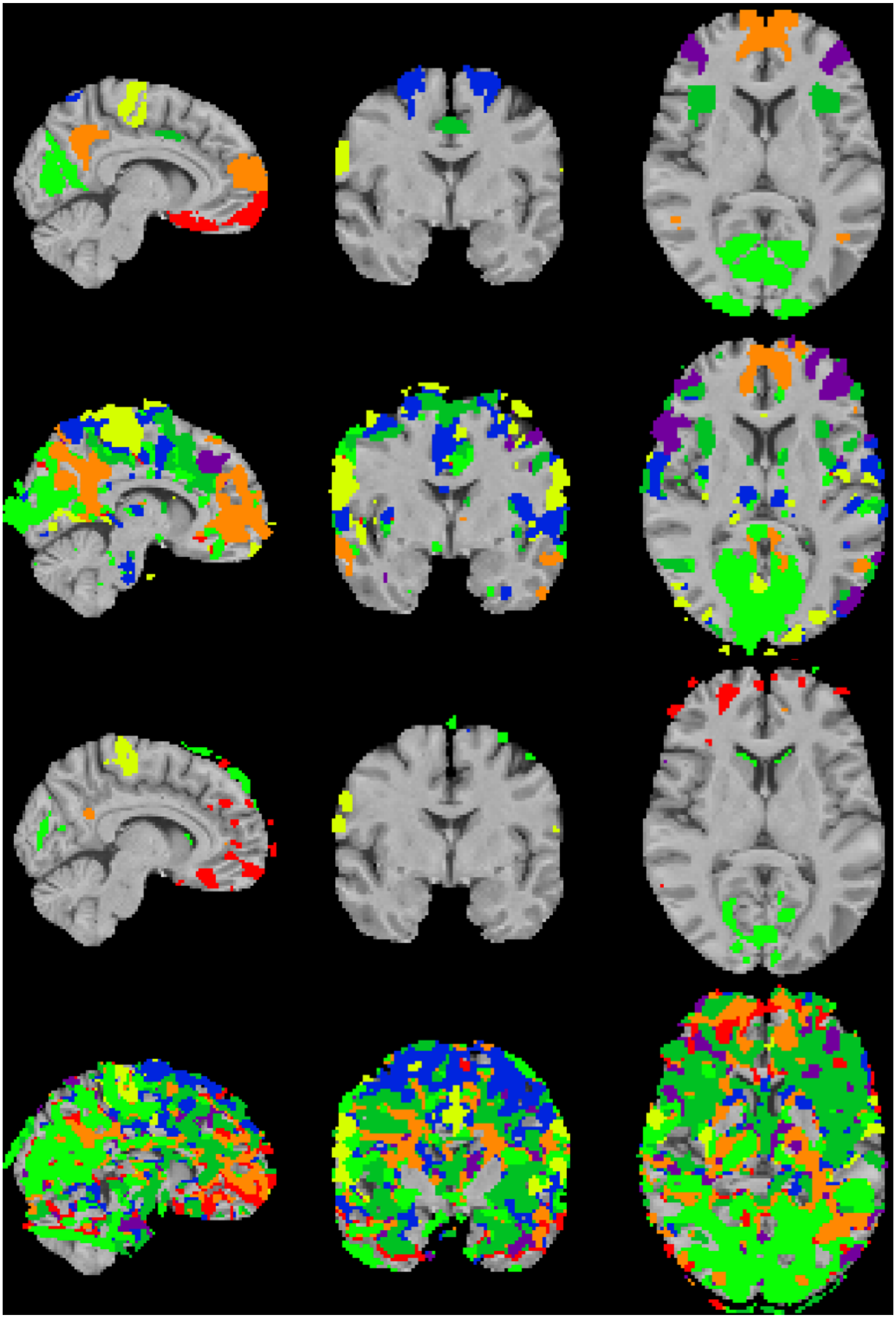

Supplementary Figure 5. *Network Identifiability of a Single Subject.* From top to bottom: (1) the network seeds derived from Brainnetome and Yeo 7-network template; (2) example network identifiability from data denoised by CICADA; (3) network identifiability from the same data but denoised with classical 8-parameter regression; (4) the same data without any denoising applied. Overall, better denoised data will result in network connectivity that is easier to visually identify and has strong and broad overlap with the network seeds.

1.4 Manual CICADA Methods

Manual CICADA methods are similar to Automatic CICADA. In short, Manual CICADA will use a user-adjusted "IC_auto_checker.csv" file – where the "SignalLabel" column is edited and then the file is renamed as "IC_manual_checker.csv" – to perform manual IC denoising. This is accomplished with a greatly shortened basescript two (which reperforms nonaggressive denoising) and the same basescript three as in Automatic CICADA. More details on implementing this can be found in the user guide on the Github. The general flow of the Manual CICADA Pipeline is also displayed in the figure below.

Manual CICADA Pipeline:

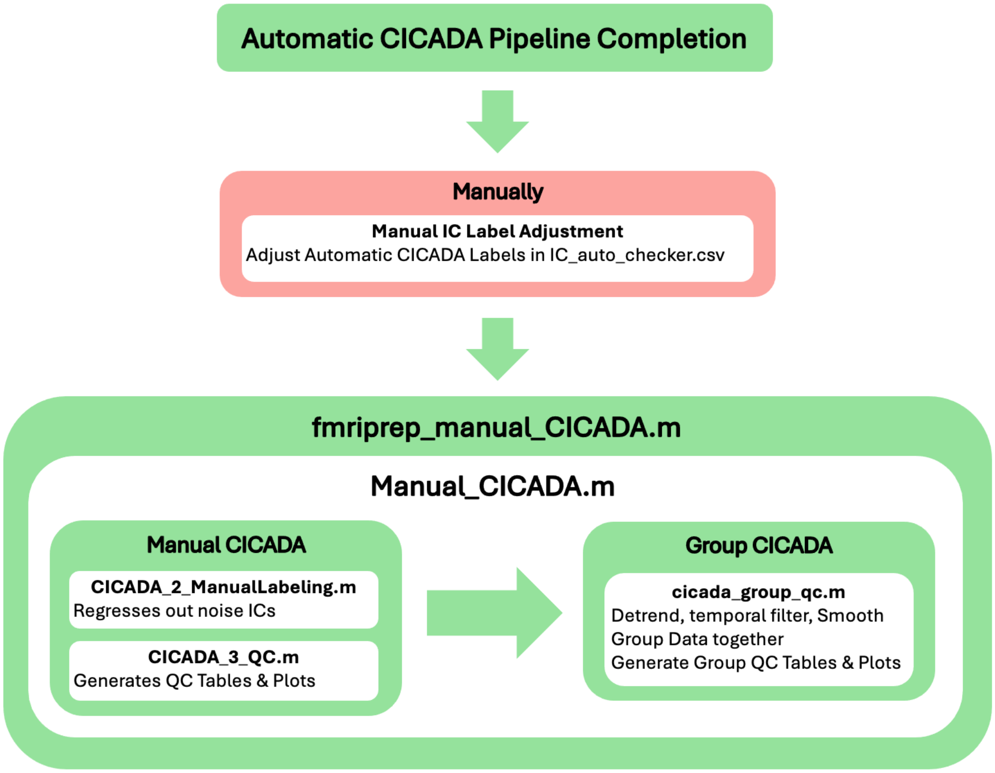

Supplementary Figure 6. *Script Flow for Manual CICADA Pipeline.* Of note, Manual_CICADA.m is sufficient to run the full Manual CICADA pipeline. The fmriprep_manual_CICADA.m may just offer an easier method to implement the Automatic CICADA pipeline for datasets that have been preprocessed with fmriprep. Automatic CICADA must be completed first to generate the IC_auto_checker.csv.

1.5 Group CICADA Methods

Group CICADA is performed through the cicada_group_qc.m function.

1.5.1 Group CICADA: Cicada_group_qc.m

Broadly, Cicada_group_qc.m performs group level adjustments, QC analyses, and prepares the data for statistical analyses. First, the function copies over all individual QC comparison plots originally generated by Automatic/Manual CICADA to one folder. This allows for easy QC plot comparison across images. Additionally, Cicada_group_qc.m copies over and combines all the individual data together into a single folder. For each image, the function uses user-defined inputs to determine if the current image should be entirely excluded or if Manual CICADA data (if it exists) should be used instead of Automatic CICADA. Cicada_group_qc.m also similarly loads the original data for each image (before denoising) and the 8p denoised image for QC comparison. Next, the specified detrending, bandpass filtering, smoothing, and voxel-wise intensity normalization of the CICADA, 8p, and original data is performed.

Following the processing of CICADA, 8p, and the original data, the relevant QC information for group QC for each of the three images are calculated. This is accomplished in several steps. First, commonly used QC cut-off values are calculated. For example, Group CICADA calculates the median and mean FD values, the %FD > 0.2 mm, if there are any FD values > 5 mm, the median DVARS, and the mean RMSD. Next, correlations within each CICADA noise profile (Edge, FD, DVARS, Outbrain, WMCSF, CSF, notGM, Suscept) and within GM are calculated in the same manner as CICADA_3_QC.m. Altogether, Group CICADA continues to calculate potentially relevant QC values and images. This includes, but is not limited to, a ratio of the gray matter mean temporal variance divided by the "notGM" (area outside of GM) temporal variance, a signal and noise IC spatial overlap image, what proportion of gray matter is covered by signal ICs, what proportion of signal ICs is found within the gray matter, a dice coefficient of gray matter and signal IC overlap, and the number and percent of ICs labeled as signal. All relevant QC calculations are then combined into relevant tables.

From there, Group CICADA performs a few last steps to aid in QC analyses. In short, this involves concatenating QC measures across images and calculating potential outliers. QC measures are marked as outliers if they are three scaled median absolute deviations ("MAD") from the median ("MAD outlier") in a given direction. Altogether, CICADA outliers are CICADA denoised data in which any of the following are true:

1. The ratio of the average GMO, S, or PSO of signal-labeled ICs to all ICs is < 1
2. The ratio of the average motion correlations (FD or DVARS) of signal-labeled ICs to all ICs is > 1
3. If the image is more than three scaled median absolute deviations below the median in any of the following measures:
    a. GM to signal-labeled IC dice coefficient
    b. The mean variance ratio of voxels within the GM to notGM masks
    c. Power Spectrum Overlap
    d. The ratio of BOLD frequency power (0.008 - 0.15 Hz) to High Frequency (> 0.15 Hz)

e. The number of ICs labeled as signal (or if less than 3 ICs are signal-labeled)

4. If the image the mean absolute correlation between gray matter voxels and either FD or DVARS ≥ 0.15 (as this is typically when the QC plots for these measures begins to look poor visually).

Group CICADA will also label conservative outliers (where either the mean FD > 0.25 mm, the %FD > 0.2mm is > 20%, or any FD > 5 mm) and liberal outliers (mean FD > 0.55 mm). The cut-offs for conservative and liberal outliers are adapted from Satterthwaite et al. 2013 (Satterthwaite et al., 2013). Next, Group CICADA saves a group QC table (containing all calculated QC data for all images, including the different types of outliers [conservative, liberal, CICADA]; Supplementary Figure 7), a group QC correlation table (containing the sampled noise profile correlations), a Group QC plot (same as the ones from CICADA_3_QC.m but for the whole group instead of per image; Supplementary Figure 8), and stacks the network identifiability images into a 4D NIfTI file to allow for easy network comparisons for each subject (see basescript 3 section). Group MELODIC is also run to generate group-level ICs, assisting evaluation of denoising success. The resulting ICs that best match each of the seven networks (Medial Visual, Sensory Motor, Dorsal Attention, Ventral Attention, FrontoParietal, Default Mode Network) from the brain network image are also returned. This is determined, in short, by sorting each IC by their dice coefficient to each network, and then testing, in order, if including the IC overall increases the resulting total dice coefficient. Altogether, this could help users better evaluate the success of the denoising in capturing different common brain networks. Other potentially helpful output files include signal_funcmasks (displays a mask of all retained signal ICs for each subject), and signal_noise_overlaps (displays regions across the whole group that were generally retained as signal vs discarded as noise) among a few others. Please see the Github and associated code to see how these are created. This concludes Group CICADA. Following QC analysis, users could, for example, use the image_names.txt in the Group CICADA folder to select the CICADA-denoised data and perform statistical analyses.

	A	B	C	D	E	F	G	H	I	J	K	CF	CG	CH	CK
1	image_path	image_names	subject	session	task	manually_ad	meanRMS	median_FD	mean_FD	Percent_FD_	AnyFD_gt_5m	cicada_outli	liberal_outlie	conservative_outliers	
2	/Volumes/Vecto	sub-102_ses-01	sub-102	ses-01	rest	0	0.15709468	0.23296021	0.25991916	58.8628763	0	0	0	1	
3	/Volumes/Vecto	sub-103_ses-01	sub-103	ses-01	rest	0	0.22381554	0.31463121	0.38060767	82.9431438	0	0	0	1	
4	/Volumes/Vecto	sub-105_ses-01	sub-105	ses-01	rest	0	0.09518034	0.147539	0.16437586	27.4247492	0	0	0	1	
5	/Volumes/Vecto	sub-106_ses-01	sub-106	ses-01	rest	0	0.11143287	0.16889714	0.19245636	31.4381271	0	0	0	1	
6	/Volumes/Vecto	sub-108_ses-01	sub-108	ses-01	rest	0	0.16542987	0.23922726	0.24883743	69.2307692	0	0	0	1	
7	/Volumes/Vecto	sub-109_ses-01	sub-109	ses-01	rest	0	0.05195827	0.0891471	0.09994078	3.67892977	0	0	0	0	
8	/Volumes/Vecto	sub-112_ses-01	sub-112	ses-01	rest	0	0.13907891	0.14793178	0.22724375	40.1337793	0	0	0	1	
9	/Volumes/Vecto	sub-114_ses-01	sub-114	ses-01	rest	0	0.23111283	0.3567336	0.38600778	90.9698997	0	0	0	1	
10	/Volumes/Vecto	sub-115_ses-01	sub-115	ses-01	rest	0	0.20663996	0.2791024	0.30343204	81.270903	0	0	0	1	
11	/Volumes/Vecto	sub-116_ses-01	sub-116	ses-01	rest	0	0.08097973	0.10632835	0.13728668	15.0501672	0	0	0	0	
12	/Volumes/Vecto	sub-117_ses-01	sub-117	ses-01	rest	0	0.15336974	0.2292912	0.23236068	59.5317726	0	0	0	1	
13	/Volumes/Vecto	sub-118_ses-01	sub-118	ses-01	rest	0	0.37132293	0.5445606	0.58648467	96.9899666	0	0	1	1	
14	/Volumes/Vecto	sub-121_ses-01	sub-121	ses-01	rest	0	0.36492196	0.501179	0.62937291	97.3244147	0	0	1	1	
15	/Volumes/Vecto	sub-122_ses-01	sub-122	ses-01	rest	0	0.2646363	0.34261068	0.40203972	88.9632107	0	0	0	1	
16	/Volumes/Vecto	sub-125_ses-01	sub-125	ses-01	rest	0	0.19243384	0.2640714	0.30270028	70.2341137	0	0	0	1	
17	/Volumes/Vecto	sub-126_ses-01	sub-126	ses-01	rest	0	0.2132036	0.3054446	0.31460983	85.9531773	0	0	0	1	
18	/Volumes/Vecto	sub-128_ses-01	sub-128	ses-01	rest	0	0.16488954	0.27203745	0.28302175	68.8963211	0	0	0	1	
19	/Volumes/Vecto	sub-129_ses-01	sub-129	ses-01	rest	0	0.15417627	0.22520083	0.22869812	60.2006689	0	0	0	1	

Supplementary Figure 7. *Group QC Table.* A truncated version of a CICADA-generated Group QC Table is shown here for reference. This table assists in QC, general data examination, and for retrieving data for analyses.

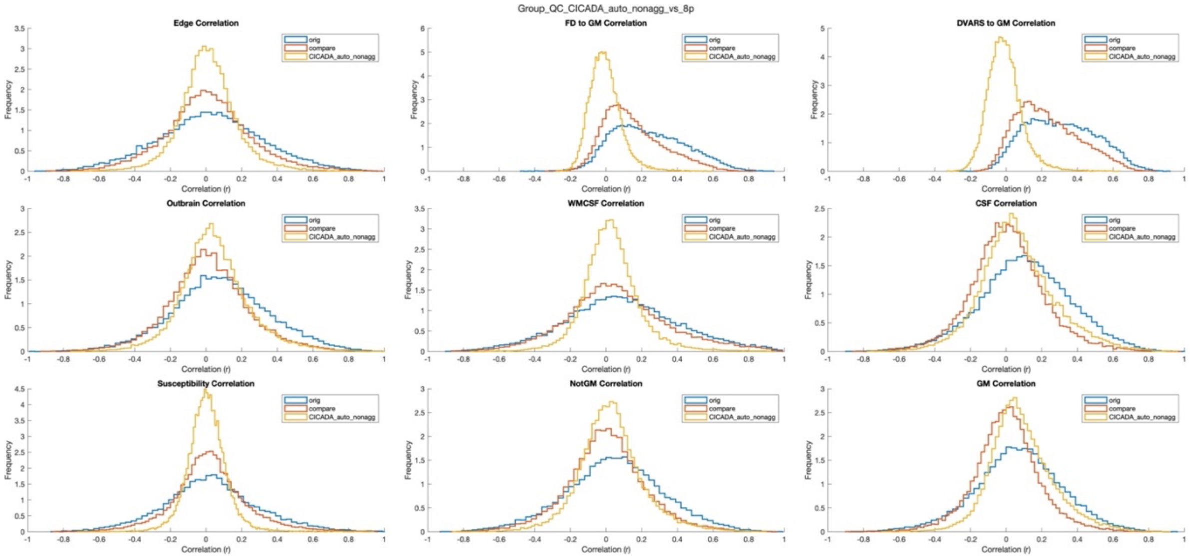

Supplementary Figure 8. *Noise Profile Correlation Histograms from Group CICADA.* These plots assist in QC analyses and can be used to compare CICADA to both the original data and other forms of denoising across a whole dataset. In this example, CICADA is compared to 8p denoising as detailed in the manuscript.

2. Additional Methods Details

Additional methodological clarifications are detailed below.

2.1 IC Classification Accuracy Parameters

Noise sensitivity (NS), noise predictive value (NPV), signal sensitivity (SS), and signal predictive value (SPV) are calculated as follows:

$$NS = \frac{TN}{TN + FS} \qquad NPV = \frac{TN}{TN + FN}$$

$$SS = \frac{TS}{TS + FN} \qquad SPV = \frac{TS}{TS + FS}$$

Where TN, “true noise,” and TS, “true signal,” are the number of ICs that the denoising method correctly classified as noise and signal, respectively. Similarly, FS, “false signal,” and FN, “false noise,” are the number of ICs that the denoising method incorrectly classified as noise and signal, respectively.

Overall accuracy (OA) is calculated as follows:

$$OA = \frac{TN\ + TS}{Total\_ICs}$$

Where *Total_ICs* are the total number of ICs for the given image. Of note, *OA* provides an easily interpretable final score but can be inappropriately biased when the class distribution (the number of ICs that are *TN* vs *TS*) is imbalanced.

Finally, the signal *F* score is calculated as follows:

$$F_s = 2\frac{SPV * SS}{SPV + SS}$$

While perhaps less interpretable than *OA*, *F* also provides a final score without being inappropriately biased by an imbalanced class distribution. Given that fMRI ICA typically has a strongly imbalanced class distribution (i.e., the *TN*:*TS* ratio is high), *F* is likely a better predictor of performance than *OA* in fMRI IC classification accuracy analysis.

3. Other CICADA Results

Additional results not included in the manuscript are described below.

3.1 Manuscript P-Values From Analyses

Tables reporting the p-values from the analyses presented in the manuscript (sections 3.1-3.4 in main manuscript) are provided below.

Supplementary Table 1. *IC Classification Accuracy Significance*

	High-Motion Task			High-Motion Rest			Low-Motion Rest		
	CICADA vs. MIRC	CICADA vs. FIX	CICADA vs. AROMA	CICADA vs. MIRC	CICADA vs. FIX	CICADA vs. AROMA	CICADA vs. MIRC	CICADA vs. FIX	CICADA vs. AROMA
	Δ% (p)	Δ% (p)	Δ% (p)	Δ% (p)	Δ% (p)	Δ% (p)	Δ% (p)	Δ% (p)	Δ% (p)
NS	-0.5 (0.027)	**4.5 (<0.001)**	**8.0 (<0.001)**	-0.4 (0.037)	**9.3 (<0.001)**	**8.8 (<0.001)**	**-1.2 (0.001)**	**7.2 (<0.001)**	**28.6 (<0.001)**
NPV	-0.8 (0.465)	-1.6 (0.017)	0.9 (0.081)	0.0 (0.935)	**-0.8 (0.002)**	0.4 (0.511)	0.7 (0.044)	0.0 (0.727)	**8.3 (<0.001)**
SS	-0.5 (0.778)	-3.7 (0.086)	5.4 (0.078)	0.7 (0.896)	-2.7 (0.088)	3.4 (0.277)	2.9 (0.023)	0.8 (0.737)	**24.1 (<0.001)**
SPV	-3.8 (0.053)	**13.7 (<0.001)**	**24.7 (<0.001)**	-4.9 (0.017)	**27.0 (<0.001)**	**25.2 (<0.001)**	**-4.2 (<0.001)**	**15.6 (<0.001)**	**48.5 (<0.001)**
OA	-1.0 (0.080)	**2.3 (0.006)**	**6.9 (<0.001)**	-0.4 (0.038)	**7.2 (<0.001)**	**7.9 (<0.001)**	-0.4 (0.627)	**5.4 (<0.001)**	**27.4 (<0.001)**
F_S	-2.5 (0.089)	**6.3 (0.014)**	**16.8 (<0.001)**	**-2.8 (0.014)**	**17.1 (<0.001)**	**19.7 (<0.001)**	-0.8 (0.989)	**9.6 (<0.001)**	**41.7 (<0.001)**

Significance was determined by signed rank tests of subject-level mean accuracy differences between CICADA and each other IC-based method (FIX, ICA-AROMA) as well as manual individual rater classification (MIRC). Significant differences, following Bonferroni correction for 3 comparisons ($p < 0.017$), are bolded. A positive Δ%, if significant, suggests that CICADA performed better than the compared method. Abbreviations: Δ%: Percent difference calculated by subtracting the given method's accuracy percentage from CICADA's (greater positive values indicate that CICADA performed better); NS: Noise sensitivity; NPV: Noise predictive value; SS: Signal sensitivity; SPV: Signal predictive value; OA: Overall Accuracy; F_S: signal F1 score; MIRC: Manual Individual Rater Classification.

Supplementary Table 2. *QC Benchmarks Significance*

	High-Motion Task				High-Motion Rest				Low-Motion Rest			
	CICADA vs. FIX	CICADA vs. AROMA	CICADA vs. 8p	CICADA vs. Original	CICADA vs. FIX	CICADA vs. AROMA	CICADA vs. 8p	CICADA vs. Original	CICADA vs. FIX	CICADA vs. AROMA	CICADA vs. 8p	CICADA vs. Original
	Δr (p)	Δr (p)	Δr (p)	Δr (p)	Δr (p)	Δr (p)	Δr (p)	Δr (p)	Δr (p)	Δr (p)	Δr (p)	Δr (p)
QC-FC	**-2.07 (<0.001)**	0.14 (0.312)	**-7.39 (<0.001)**	**-6.24 (<0.001)**	**-2.44 (<0.001)**	0.04 (0.258)	**-7.28 (<0.001)**	**-10.40 (<0.001)**	**-3.96 (<0.001)**	**-2.44 (<0.001)**	0.03 (0.432)	**0.36 (<0.001)**

DD	**-1.70 (<0.001)**	-0.16 (0.406)	**-15.16 (<0.001)**	**-5.17 (<0.001)**	**-2.81 (<0.001)**	-0.44 (0.300)	**-20.51 (<0.001)**	**-7.05 (<0.001)**	-0.18 (0.642)	0.07 (0.890)	**-1.99 (0.011)**	-1.47 (0.028)
Q	0.02 (0.044)	-0.21 (0.802)	**-21.6 (<0.001)**	**3.40 (<0.001)**	0.96 (0.014)	0.11 (0.766)	**-26.49 (<0.001)**	1.03 (0.237)	**1.45 (0.001)**	**2.87 (<0.001)**	**2.73 (<0.001)**	**7.42 (<0.001)**
QC-Q	-1.06 (0.299)	-0.47 (0.117)	-16.43 (0.099)	-3.88 (0.252)	-4.11 (0.365)	4.21 (0.284)	-7.08 (0.041)	3.59 (0.732)	-9.32 (0.031)	-14.99 (0.057)	-0.77 (0.939)	-0.94 (0.879)

Significance was determined by signed rank tests between CICADA and each other method (FIX, ICA-AROMA, 8p, original data before denoising). Significant differences, following Bonferroni correction for four comparisons ($p < 0.0125$), are bolded. For QC-FC, DD, and QC-Q, a negative Δr, if significant, generally suggests that CICADA performed better than the compared method. For Q, a positive Δr, if significant, generally suggests that CICADA performed better than the compared method. Abbreviations: Δr: Mean difference in correlation (multiplied by 100 for readability); QC-FC: Quality Control (median FD) to Functional Connectivity; DD: Distance dependence; Q: Modularity; QC-Q: Quality Control (median FD) to Modularity.

Supplementary Table 3. *Loss of Temporal Degrees of Freedom Significance*

	High-Motion Task			High-Motion Rest			Low-Motion Rest		
	Manual vs. CICADA	Manual vs. FIX	Manual vs. AROMA	Manual vs. CICADA	Manual vs. FIX	Manual vs. AROMA	Manual vs. CICADA	Manual vs. FIX	Manual vs. AROMA
	Δ (p)	Δ (p)	Δ (p)	Δ (p)	Δ (p)	Δ (p)	Δ (p)	Δ (p)	Δ (p)
Fraction of Noise ICs	-0.01 (0.025)	**0.03 (<0.001)**	**0.04 (<0.001)**	-0.00 (0.717)	**0.08 (<0.001)**	**0.07 (<0.001)**	+0.00 (0.356)	**0.06 (<0.001)**	**0.17 (<0.001)**
Number of Noise ICs	-0.67 (0.039)	**2.13 (<0.001)**	**2.77 (<0.001)**	0.03 (0.852)	**8.30 (<0.001)**	**7.90 (<0.001)**	0.20 (0.372)	**3.90 (<0.001)**	**11.80 (<0.001)**

Temporal degrees of freedom loss was estimated by the number of ICs classified as noise by the given methods. Significance was determined by signed rank tests between manual classification and each IC-based denoising method (CICADA, FIX, ICA-AROMA). Significant p-values, following Bonferroni correction for 3 comparisons ($p < 0.017$), are bolded. Abbreviations: Δ: Mean difference in fraction (row 1) or number (row 2) of noise ICs; TDOF: temporal degrees of freedom.

Supplementary Table 4. *Noise Profile Significance*

	High-Motion Task				High-Motion Rest				Low-Motion Rest			
	CICADA vs. FIX	CICADA vs. AROMA	CICADA vs. 8p	CICADA vs. Original	CICADA vs. FIX	CICADA vs. AROMA	CICADA vs. 8p	CICADA vs. Original	CICADA vs. FIX	CICADA vs. AROMA	CICADA vs. 8p	CICADA vs. Original
	ΔM (p)	ΔM (p)	ΔM (p)	ΔM (p)	ΔM (p)	ΔM (p)	ΔM (p)	ΔM (p)	ΔM (p)	ΔM (p)	ΔM (p)	ΔM (p)
Edge	**-1.18 (<0.001)**	-0.49 (0.736)	**-6.66 (<0.001)**	**-12.06 (<0.001)**	**-2.42 (<0.001)**	-0.54 (0.880)	**-6.36 (<0.001)**	**-11.59 (<0.001)**	**-0.72 (<0.001)**	-0.32 (0.096)	**-2.74 (<0.001)**	**-6.88 (<0.001)**
Outbrain	**-1.21 (<0.001)**	-0.11 (0.821)	**-3.73 (<0.001)**	**-8.64 (<0.001)**	**-3.06 (<0.001)**	0.19 (0.126)	**-2.65 (0.001)**	**-7.72 (<0.001)**	**-0.58 (<0.001)**	**-1.10 (<0.001)**	**-3.13 (<0.001)**	**-4.76 (<0.001)**
Subepe	-1.57 (0.015)	**-0.95 (0.005)**	**-10.51 (<0.001)**	**-14.55 (<0.001)**	**-5.37 (<0.001)**	**-1.17 (<0.001)**	**-10.63 (<0.001)**	**-14.50 (<0.001)**	-0.05 (0.133)	**-0.29 (<0.001)**	**-0.78 (<0.001)**	**-1.49 (<0.001)**
CSF	**-1.18 (<0.001)**	0.08 (0.688)	-1.48 (0.039)	**-7.05 (<0.001)**	**-2.61 (<0.001)**	0.50 (0.050)	0.64 (0.431)	**-5.46 (<0.001)**	**-0.62 (<0.001)**	-0.35 (0.028)	**-0.91 (<0.001)**	**-2.91 (<0.001)**
Susc	-0.83 (0.067)	**-0.93 (0.009)**	**-5.78 (<0.001)**	**-11.89 (<0.001)**	**-2.03 (<0.001)**	**-1.58 (0.001)**	**-7.48 (<0.001)**	**-12.65 (<0.001)**	-0.07 (0.037)	**-0.29 (<0.001)**	**-0.90 (<0.001)**	**-2.80 (<0.001)**
NotGM	**-1.12 (<0.001)**	-0.24 (0.959)	**-4.06 (<0.001)**	**-9.32 (<0.001)**	**-2.33 (<0.001)**	-0.05 (0.258)	**-3.24 (<0.001)**	**-8.37 (<0.001)**	**-0.60 (<0.001)**	-0.30 (0.035)	**-1.44 (<0.001)**	**-4.32 (<0.001)**
FD-GM	-0.84 (0.205)	**0.54 (0.007)**	**-4.57 (<0.001)**	**-12.67 (<0.001)**	**-3.24 (<0.001)**	-2.14 (0.013)	**-10.48 (<0.001)**	**-18.60 (<0.001)**	-0.05 (0.247)	**0.15 (0.009)**	**-0.78 (<0.001)**	**-1.37 (<0.001)**
DVARS-GM	-1.35 (0.145)	**0.60 (0.002)**	**-8.74 (<0.001)**	**-17.39 (<0.001)**	**-5.40 (<0.001)**	-2.54 (0.016)	**-16.43 (<0.001)**	**-23.82 (<0.001)**	-0.04 (0.625)	**0.21 (0.008)**	**-1.06 (<0.001)**	**-1.69 (<0.001)**
DS	**2.45 (0.005)**	1.03 (0.346)	**21.87 (<0.001)**	**16.25 (<0.001)**	**7.94 (<0.001)**	2.72 (0.049)	**26.34 (<0.001)**	**17.61 (<0.001)**	**4.01 (<0.001)**	2.52 (0.042)	**14.61 (<0.001)**	**19.61 (<0.001)**

Significance was determined by signed rank tests between CICADA and each other method (FIX, ICA-AROMA, 8p, original data before denoising). Significant p-values, following Bonferroni correction for 4 comparisons ($p < 0.0125$), are bolded. For all noise profiles, a negative ΔM, if significant, suggests that CICADA performed better than the compared method. For DS, a positive ΔM, if significant, suggests that CICADA performed better than the compared method. Abbreviations: ΔM: Mean difference (multiplied by 100 for readability); Subepe: Subependymal noise profile; CSF: cerebral spinal fluid; NotGM: All the functional mask except for the gray matter; FD: framewise displacement; DVARS: derivative of root mean square VARiance over voxels; DS: denoising success.

3.2 Modularity and QC-Modularity Benchmarks

Modularity (Q) and QC-Modularity (QC-Q) are reported in some related literature. While helpful measures, Q may penalize internetwork connectivity and may wrongfully reward noise retention if the noise source is dominant and consistent. Therefore, modularity heat maps and QC-Q plots are included in the supplementary material below.

Of note, modularity aims to quantify how well independent brain networks can be identified (Sporns & Betzel, 2015). For each subject, modularity was calculated through the Louvain method for community detection with accommodation for signed data (Blondel et al., 2008). Greater modularity indicates better detection of structured and independent sub-networks. Across all subjects, the mean modularity for each method was also calculated. Overall, a greater mean modularity for a given method may suggest greater brain network identifiability.

Meanwhile, The QC-Q correlation helps assess the influence of motion on independent brain network identification (Satterthwaite et al., 2012). Across all subjects, QC-Q was calculated as the correlation between mFD and modularity. A greater QC-Q magnitude indicates a greater impact of motion on network identifiability. As such, the QC-Q magnitude for CICADA and each other denoising method was compared with a Williams paired correlation test (Williams, 1959). Overall, a smaller QC-Q magnitude may suggest greater reduction of motion impact on network identifiability.

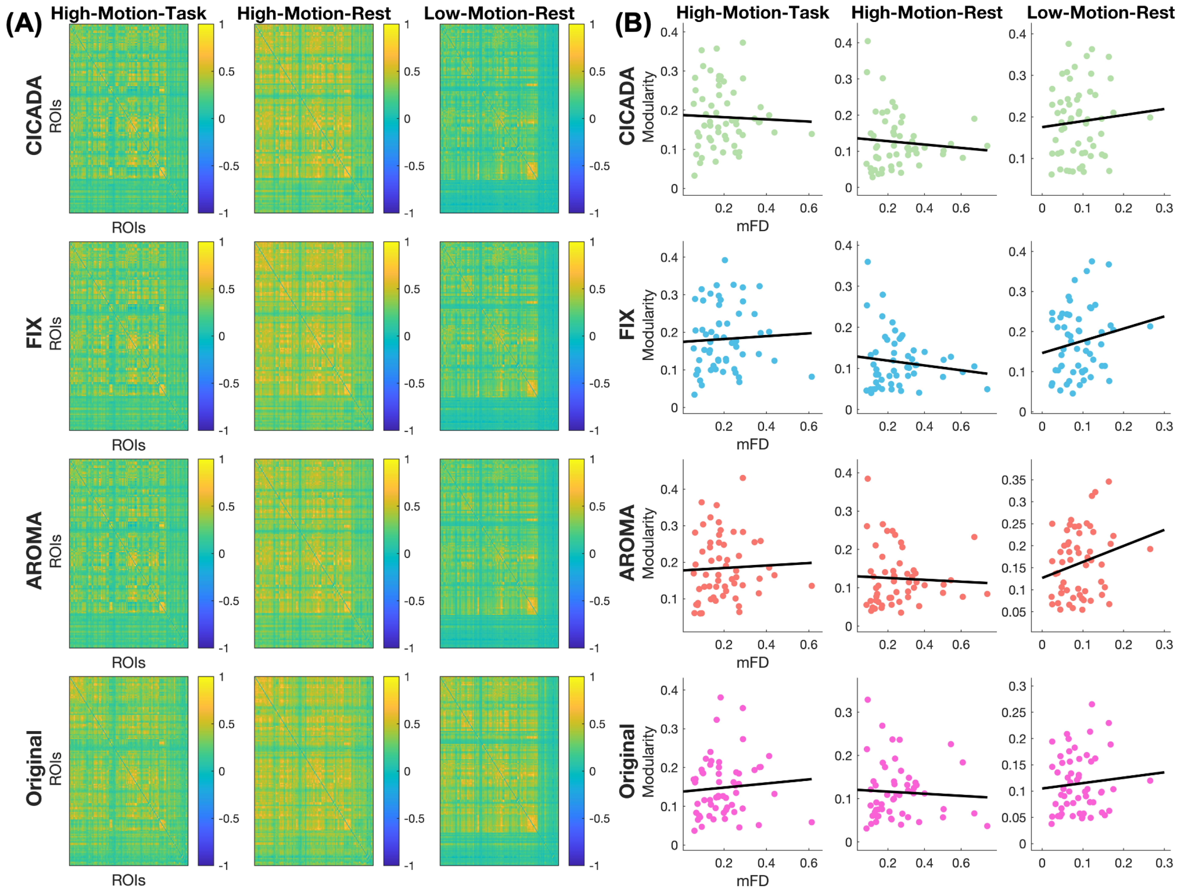

Supplementary Figure 9. Modularity (Q) *and QC-Q Plots*. (A) Heat maps of the mean functional connectivity of each ROI pair across subjects. Better denoised data will typically have more recognizable clusters of greater functional connectivity. (B) QC-Q plots demonstrating the correlation (black line) between median FD and Modularity (Q). Better denoised data will typically have lower correlation magnitudes. Abbreviations: ROI: region of interest.

3.3 Comparisons to 8p Denoising

In addition to comparing CICADA to FIX and ICA-AROMA, we also compared CICADA to 8p (simple regression of the six motion parameters + white matter + cerebral spinal fluid), where possible. The relevant figures are provided below.

In Supplementary Figure 10, both mean modularity (Q), and the mean absolute correlation of median FD to Q (QC-Q) are reported. Q, which helps measure network identifiability (Sporns & Betzel, 2015), was calculated through the Louvain method for community detection with accommodation for signed data (Blondel et al., 2008). QC-Q was calculated as the correlation between median FD and Q. Unlike all other denoising performance measures, QC-Q significance was determined by the Williams paired correlation test (Williams, 1959), instead of a Wilcoxon signed rank test (Wilcoxon, 1945), as QC-Q comparisons involve paired correlations. Both Q and QC-Q may be used to help infer network identifiability, where a greater Q and a lesser QC-Q typically indicate improved network identifiability. Of note though, between-network connectivity, prevalent in true neural signal, will reduce Q scores. Therefore, Q and QC-Q, may not be optimal measurements of network identifiability in fMRI connectivity data.

Regardless, the Q and QC-Q results show similar trends to the F_S and DS trends presented in the manuscript. CICADA appeared to significantly outperform both FIX and ICA-AROMA in network identifiability (>Q) in the low-motion dataset especially. This may suggest that CICADA is more effective in increasing network identifiability than FIX or ICA-AROMA in lower motion datasets especially. The greater Q in eight parameter denoising in the high motion datasets, meanwhile, is likely reflective of motion instead of true neural signal, as suggested by the associated larger QC-Q values. Given that F_S and DS already help evaluate neural signal retention in the manuscript, these Q and QC-Q parameters are only included in the supplementary material. Of note, all four metrics in Supplementary Figure 10 demonstrate high subject variability, especially in QC-Q. While this variability may warrant caution in interpretation, these metrics may still serve as valuable group-level benchmarks for assessing motion-related noise removal.

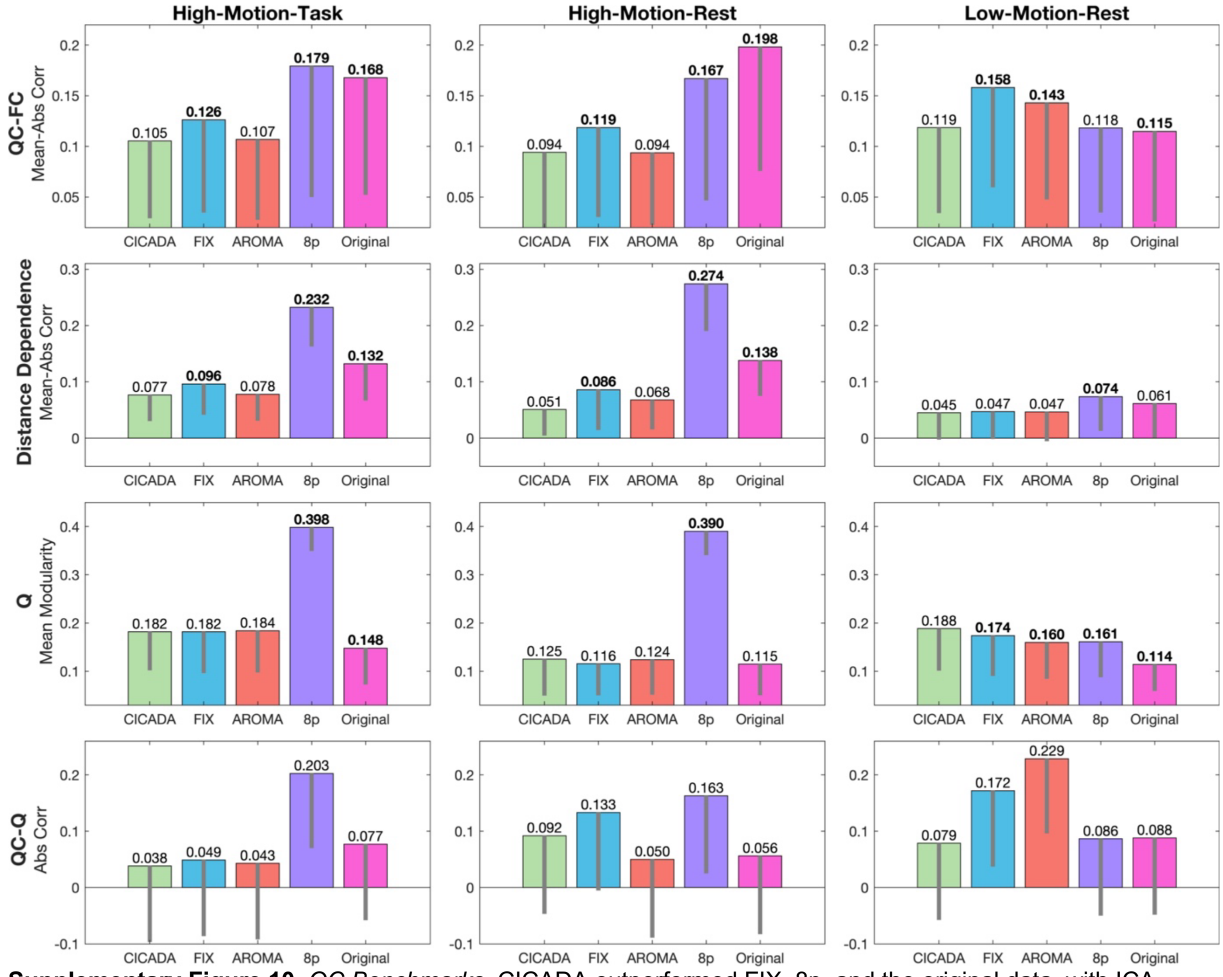

Supplementary Figure 10. *QC Benchmarks*. CICADA outperformed FIX, 8p, and the original data, with ICA-AROMA performing the closest in comparison. For QC-FC, Distance Dependence, and QC-Q, lower mean scores are typically better. For Q, higher mean scores are typically better. Bolded values denote significant differences between CICADA and each other method, Bonferroni corrected for the four comparisons ($p < 0.0125$). The gray lines depict one standard deviation below the mean in all cases except for QC-Q; as QC-Q is a single correlation value, the gray line depicts the lower bound of a 67% confidence interval to approximate the spread of a single standard deviation. Abbreviations: QC-FC: Quality control (median FD) and functional connectivity mean correlation magnitudes; Distance Dependence: Functional connectivity of region-of-interest pairs and distance mean correlation magnitudes; Q: Mean Modularity; QC-Q: Quality control (median FD) and modularity mean

correlation. Mean-Abs Corr: mean of the magnitude (absolute value) of the correlations; Abs Corr: magnitude of the correlation.

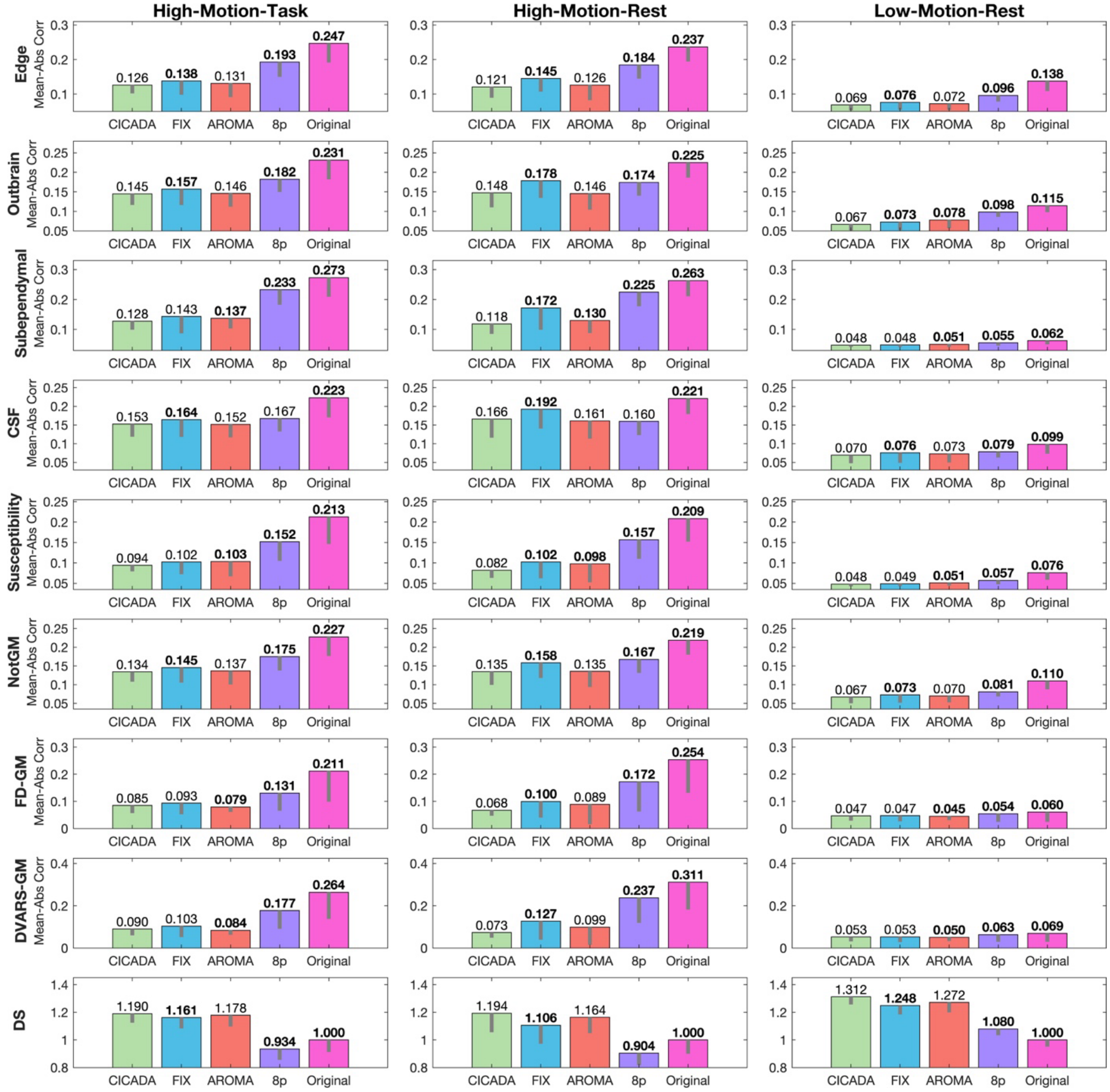

Supplementary Figure 11. *Noise Profile Correlations and Denoising Success*. CICADA performed the best across all datasets and noise profiles, and CICADA resulted in the highest denoising success. ICA-AROMA performed the most similarly to CICADA overall, especially in the high-motion datasets. FIX also performed similarly to CICADA in the low-motion-rest dataset. 8p performed significantly worse than all three ICA-based denoising methods. The gray lines depict one standard deviation below the mean in all cases except for QC-Q; as QC-Q is a single correlation value, the gray line depicts the lower bound of a 67% confidence interval to approximate the spread of a single standard deviation. Bolded values denote significant differences between CICADA and each other method, Bonferroni corrected for the four comparisons ($p < 0.0125$). Abbreviations: Mean-Abs Corr: mean magnitude of the correlations; FD: framewise displacement; DVARS: temporal Derivative of root mean square VARiance over voxels; CSF: cerebral spinal fluid; NotGM: not gray matter; DS: denoising success.

3.4 Loss of Temporal Degrees of Freedom

Loss of the temporal degrees of freedom was also approximated by the mean number of Noise ICs identified by the given method (manual denoising, CICADA, FIX, and ICA-AROMA). Both manual denoising and CICADA removed a similar number of noise ICs.

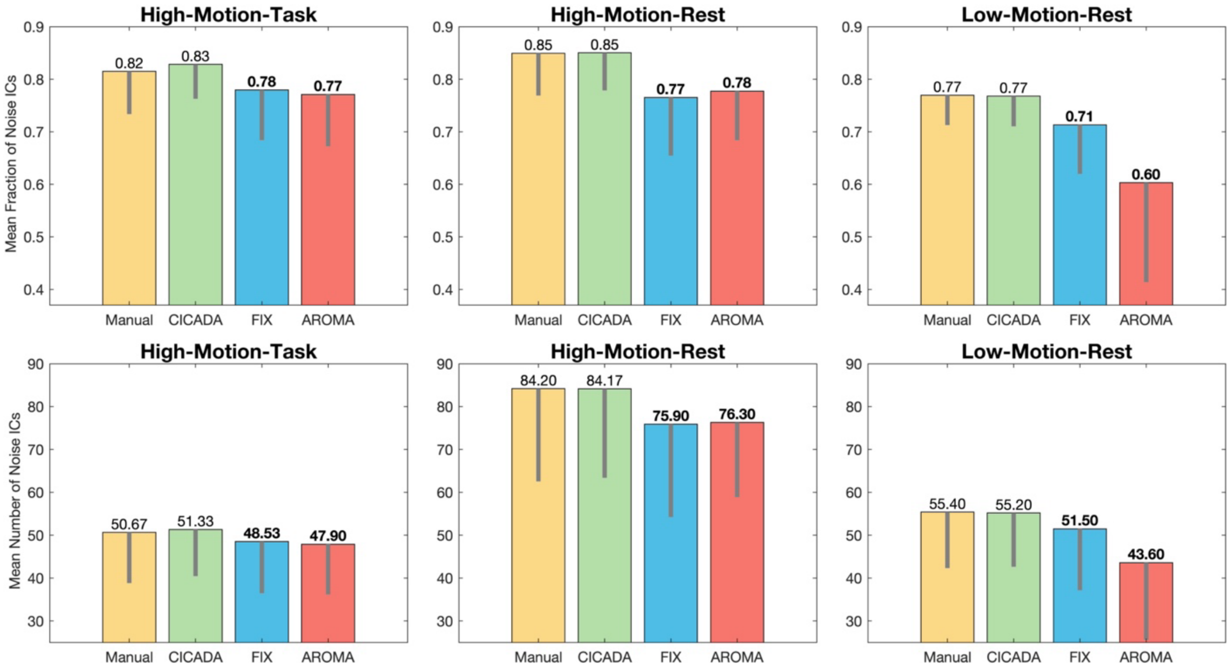

Supplementary Figure 12. *Loss of Temporal Degrees of Freedom.* Loss of Temporal Degrees of Freedom was approximated by the mean number of Noise ICs identified by the given method. The first row denotes the overall mean fraction of the ICs that were labelled as noise by the given method, while the second row denotes the overall mean number. CICADA reduced the temporal degrees of freedom similarly to manual IC classification, and more than both FIX and ICA-AROMA. The high-motion-rest dataset contained the greatest percentage of true Noise ICs compared to the other datasets. The gray lines depict one standard deviation below the mean. Bolded values denote significant differences between manual IC classification and each other method, Bonferroni corrected for the three comparisons ($p < 0.017$). Abbreviations: IC: Independent Component.

3.5 Comparing Higher Motion Multiband to Lower Motion Multiband

To preliminarily examine if the degree of participant motion impacts IC classification accuracy of CICADA and the other ICA-based classification methods, we tested for IC classification accuracy differences between higher-motion (N = 6, mFD = 0.149 $\pm$ 0.020 $[\mu \pm \sigma]$) and lower-motion (N = 24, mFD = 0.074 $\pm$ 0.020 $[\mu \pm \sigma]$) participants from the original low-motion multiband dataset. Higher-motion participants were characterized by Sattherwaite et al. guidelines for conservative outliers (i.e., where either the mean FD > 0.25 mm, the %FD > 0.2mm is > 20%, or any FD > 5 mm) (Satterthwaite et al., 2013). We also calculated the correlations of median FD to IC classification accuracy for the whole group.

The results of both tests can be seen in the tables below. In summary, CICADA accuracy was not significantly different between higher-motion and lower-motion participants in the multiband dataset, nor was motion (median framewise displacement) significantly correlated to any accuracy measure. Only FIX resulted in p-values < 0.05: higher-motion participants demonstrated lower signal sensitivity (p = 0.017), and motion

was negatively correlated with signal sensitivity accuracy (p = 0.023). Altogether this supports CICADA's use in both higher- and lower-motion multiband datasets.

Supplementary Table 5. Multiband Accuracy Comparison of Higher-Motion vs Lower-Motion

	CICADA			MIRC			FIX			ICA-AROMA		
	Higher-Motion	Lower-Motion		Higher-Motion	Lower-Motion		Higher-Motion	Lower-Motion		Higher-Motion	Lower-Motion	
	M±SD	*M±SD*	*p*	*M±SD*	*M±SD*	*p*	*M±SD*	*M±SD*	*p*	*M±SD*	*M±SD*	*p*
NS	0.990±0.02	0.983±0.02	0.481	0.998±0.00	0.996±0.01	0.482	0.921±0.14	0.911±0.06	0.789	0.768±0.20	0.681±0.20	0.341
NPV	0.982±0.03	0.989±0.02	0.485	0.983±0.02	0.980±0.01	0.717	0.980±0.02	0.989±0.01	0.229	0.888±0.06	0.908±0.08	0.554
SS	0.952±0.08	0.955±0.09	0.946	0.904±0.12	0.931±0.05	0.391	0.872±0.16	0.965±0.05	**0.017**	0.571±0.23	0.749±0.20	0.069
SPV	0.940±0.12	0.946±0.08	0.872	0.985±0.04	0.987±0.03	0.891	0.823±0.20	0.780±0.13	0.519	0.450±0.25	0.462±0.16	0.881
OA	0.978±0.03	0.978±0.03	0.997	0.984±0.02	0.981±0.01	0.639	0.926±0.09	0.924±0.04	0.925	0.742±0.13	0.694±0.14	0.449
F_S	0.940±0.08	0.947±0.07	0.834	0.941±0.09	0.957±0.03	0.424	0.826±0.14	0.855±0.08	0.503	0.468±0.18	0.544±0.12	0.222

p-values < 0.05 are bolded. Higher-Motion group is defined by conservative outlier thresholds (see text). Abbreviations: NS: noise sensitivity; NPV: noise predictive value; SS: signal sensitivity; SPV: signal predictive value; OA: overall accuracy; F_S: signal F-score; mFD: median framewise displacement.

Supplementary Table 6. Correlation of Motion (mFD) to IC Classification Accuracy in low-motion multiband dataset

	CICADA	MIRC	FIX	ICA-AROMA
	r (p)	*r (p)*	*r (p)*	*r (p)*
NS	0.19 (0.312)	-0.12 (0.511)	0.12 (0.536)	0.23 (0.216)
NPV	-0.19 (0.313)	0.12 (0.522)	-0.26 (0.169)	0.10 (0.600)
SS	-0.06 (0.738)	-0.13 (0.509)	-0.41 (**0.023**)	-0.09 (0.627)
SPV	0.02 (0.924)	-0.20 (0.285)	0.18 (0.350)	0.11 (0.576)
OA	0.00 (0.982)	0.04 (0.839)	0.10 (0.600)	0.30 (0.102)
F_S	-0.05 (0.792)	-0.18 (0.349)	-0.02 (0.897)	0.08 (0.656)

p-values < 0.05 are bolded. Abbreviations: NS: noise sensitivity; NPV: noise predictive value; SS: signal sensitivity; SPV: signal predictive value; OA: overall accuracy; F_S: signal F-score; mFD: median framewise displacement.

3.6 Comparing to Manual ICA in Manual Subsets

QC benchmarks and noise profiles for all methods (including CICADA) within the manual subsets (N = 30 for each) were also compared to manual ICA (inter-rater majority vote of the three raters in MIRC). Significant differences of a given method compared to manual ICA (corrected for multiple comparisons) are bolded in the figures. Overall, CICADA performed similarly to manual ICA.

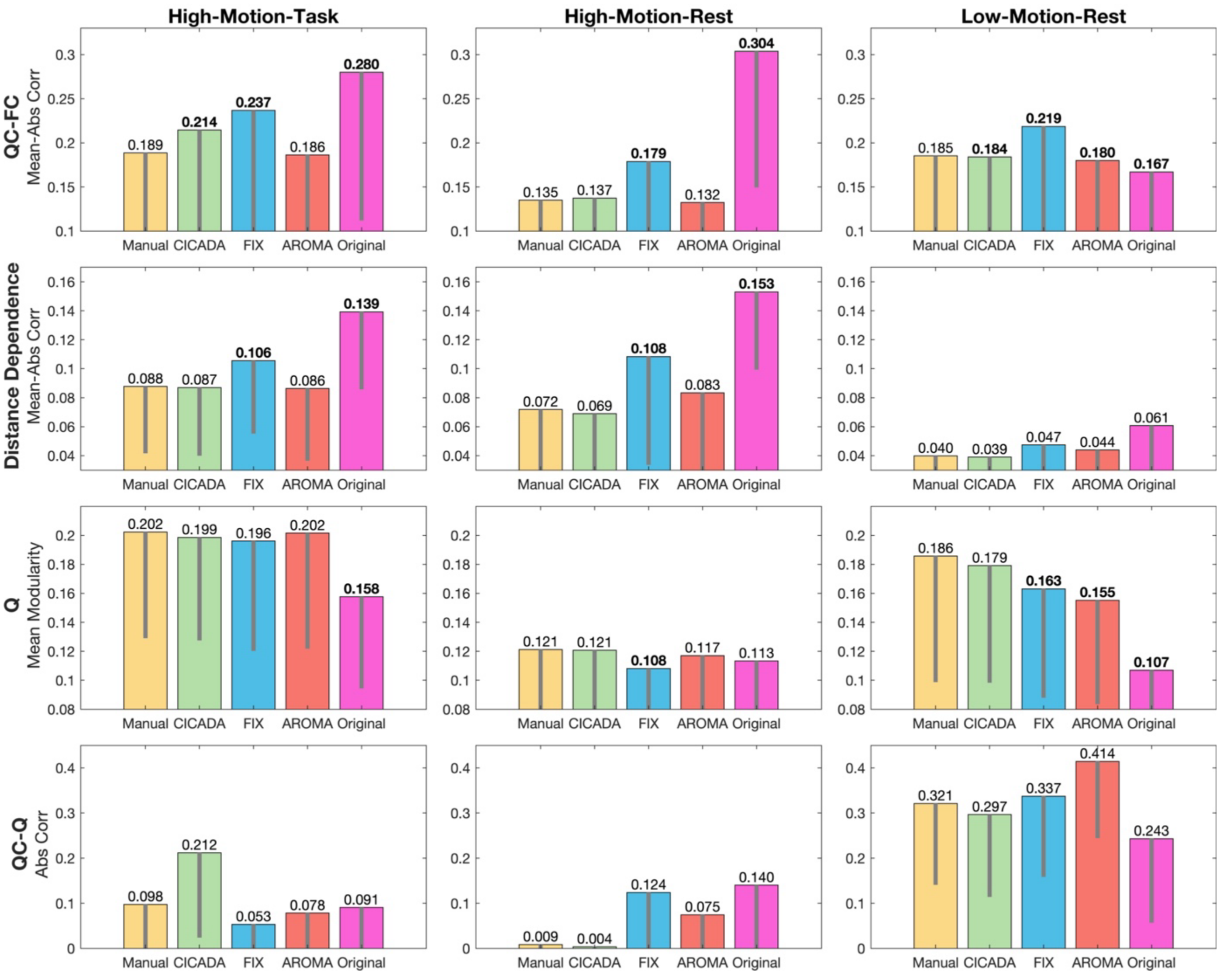

Supplementary Figure 13. *QC Benchmarks Compared To Manual ICA in Manual Subsets*. In the manual subsets (N = 30 per group), CICADA and ICA-AROMA performed the most similarly to manual ICA in the QC benchmarks. For QC-FC, Distance Dependence, and QC-Q, lower mean scores are typically better. For Q, higher mean scores are typically better. Bolded values denote significant differences between Manual ICA and each other method (including CICADA), Bonferroni corrected for the four comparisons ($p < 0.0125$). The gray lines depict one standard deviation below the mean in all cases except for QC-Q; as QC-Q is a single correlation value, the gray line depicts the lower bound of a 67% confidence interval to approximate the spread of a single standard deviation. Abbreviations: QC-FC: Quality control (median FD) and functional connectivity mean correlation magnitudes; Distance Dependence: Functional connectivity of region-of-interest pairs and distance mean correlation magnitudes; Q: Mean Modularity; QC-Q: Quality control (median FD) and modularity mean correlation. Mean-Abs Corr: mean of the magnitude (absolute value) of the correlations; Abs Corr: magnitude of the correlation.

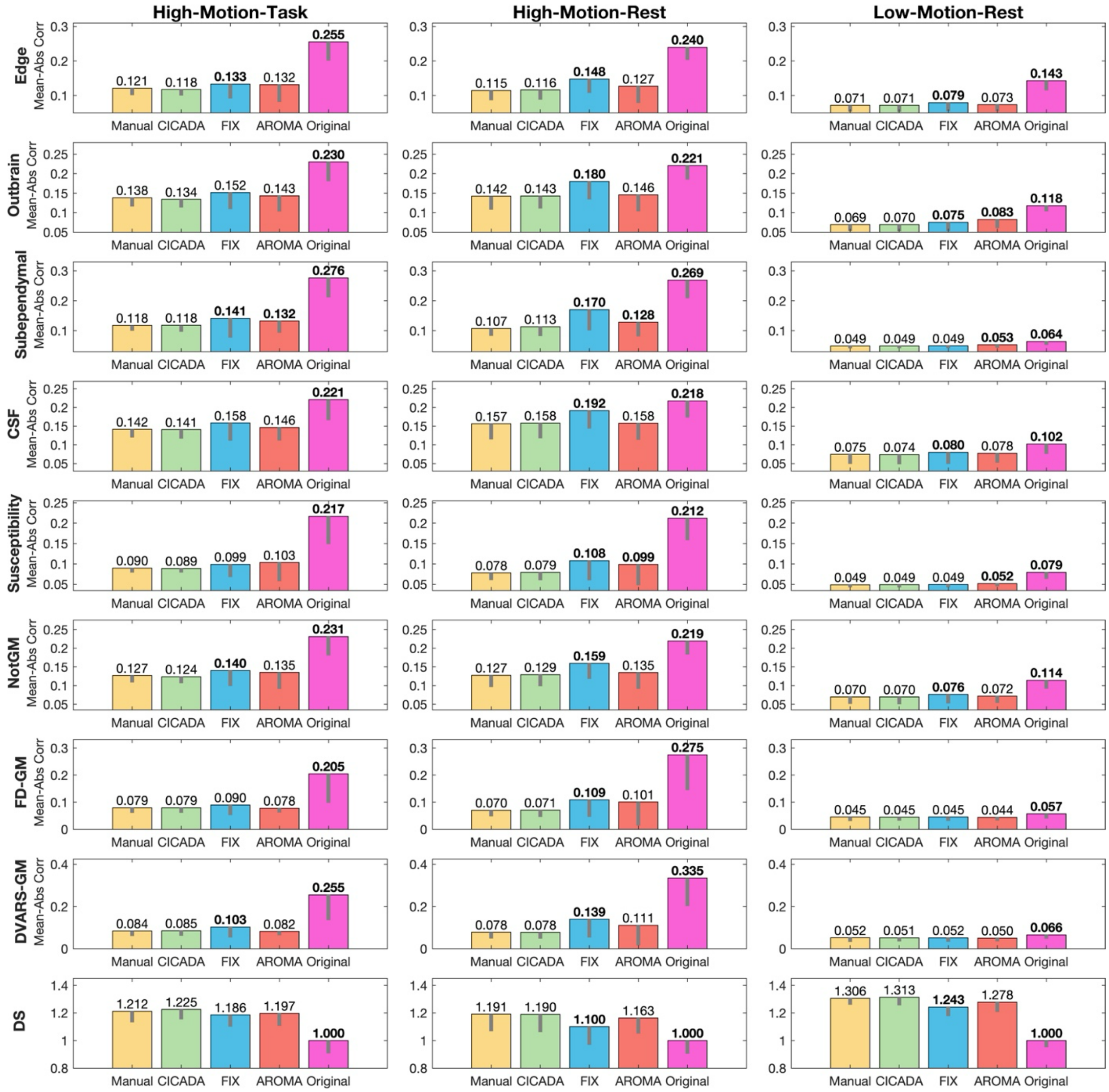

Supplementary Figure 14. *Noise Profile Correlations and Denoising Success Compared to Manual ICA in Manual Subsets*. In the manual subsets (N = 30 per group), CICADA did not significantly differ from manual ICA in any of the noise profiles or Denoising Success (DS) parameter. ICA-AROMA performed the most similarly to CICADA overall, especially in the high-motion datasets. FIX also performed similarly to CICADA in the low-motion-rest dataset. The gray lines depict one standard deviation below the mean in all cases except for QC-Q; as QC-Q is a single correlation value, the gray line depicts the lower bound of a 67% confidence interval to approximate the spread of a single standard deviation. Bolded values denote significant differences between Manul ICA and each other method, Bonferroni corrected for the four comparisons ($p < 0.0125$). Abbreviations: Mean-Abs Corr: mean magnitude of the correlations; FD: framewise displacement; DVARS: temporal Derivative of root mean square VARiance over voxels; CSF: cerebral spinal fluid; NotGM: not gray matter; DS: denoising success.

Supplementary Material References:

Beckmann, C. F., & Smith, S. M. (2004). Probabilistic independent component analysis for functional magnetic resonance imaging. *IEEE Transactions on Medical Imaging*, *23*(2), 137–152. https://doi.org/10.1109/TMI.2003.822821

Blondel, V. D., Guillaume, J.-L., Lambiotte, R., & Lefebvre, E. (2008). Fast unfolding of communities in large networks. *Journal of Statistical Mechanics: Theory and Experiment*, *2008*(10), P10008. https://doi.org/10.1088/1742-5468/2008/10/P10008

Fan, L., Li, H., Zhuo, J., Zhang, Y., Wang, J., Chen, L., Yang, Z., Chu, C., Xie, S., Laird, A. R., Fox, P. T., Eickhoff, S. B., Yu, C., & Jiang, T. (2016). The Human Brainnetome Atlas: A New Brain Atlas Based on Connectional Architecture. *Cerebral Cortex (New York, N.Y.: 1991)*, *26*(8), 3508–3526. https://doi.org/10.1093/cercor/bhw157

Fonov, V. S., Evans, A. C., McKinstry, R. C., Almli, C. R., & Collins, D. L. (2009). Unbiased nonlinear average age-appropriate brain templates from birth to adulthood. *Neuroimage*, *47*, S102.

Griffanti, L., Douaud, G., Bijsterbosch, J., Evangelisti, S., Alfaro-Almagro, F., Glasser, M. F., Duff, E. P., Fitzgibbon, S., Westphal, R., Carone, D., Beckmann, C. F., & Smith, S. M. (2017). Hand classification of fMRI ICA noise components. *Neuroimage*, *154*, 188–205.

Guzmán-Vélez, E., Diez, I., Schoemaker, D., Pardilla-Delgado, E., Vila-Castelar, C., Fox-Fuller, J. T., Baena, A., Sperling, R. A., Johnson, K. A., Lopera, F., Sepulcre, J., & Quiroz, Y. T. (2022). Amyloid-β and tau pathologies relate to distinctive brain dysconnectomics in preclinical autosomal-dominant Alzheimer’s disease.

Proceedings of the National Academy of Sciences of the United States of America, *119*(15), e2113641119. https://doi.org/10.1073/pnas.2113641119

Power, J. D., Barnes, K. A., Snyder, A. Z., Schlaggar, B. L., & Petersen, S. E. (2012). Spurious but systematic correlations in functional connectivity MRI networks arise from subject motion. *NeuroImage*, *59*(3), 2142–2154. https://doi.org/10.1016/j.neuroimage.2011.10.018

Satterthwaite, T. D., Elliott, M. A., Gerraty, R. T., Ruparel, K., Loughead, J., Calkins, M. E., Eickhoff, S. B., Hakonarson, H., Gur, R. C., Gur, R. E., & Wolf, D. H. (2013). An improved framework for confound regression and filtering for control of motion artifact in the preprocessing of resting-state functional connectivity data. *Neuroimage*, *64*, 240–256.

Satterthwaite, T. D., Wolf, D. H., Loughead, J., Ruparel, K., Elliott, M. A., Hakonarson, H., Gur, R. C., & Gur, R. E. (2012). Impact of in-scanner head motion on multiple measures of functional connectivity: Relevance for studies of neurodevelopment in youth. *NeuroImage*, *60*(1), 623–632. https://doi.org/10.1016/j.neuroimage.2011.12.063

Sporns, O., & Betzel, R. F. (2015). Modular Brain Networks. *Annual Review of Psychology*, *67*, 613. https://doi.org/10.1146/annurev-psych-122414-033634

Wilcoxon, F. (1945). Individual Comparisons by Ranking Methods. *Biometrics Bulletin*, *1*(6), 80–83. https://doi.org/10.2307/3001968

Williams, E. (1959). *Regression analysis* (Vol. 14). Wiley. https://cir.nii.ac.jp/crid/1130282272046092416

Yeo, B. T. T., Krienen, F. M., Sepulcre, J., Sabuncu, M. R., Lashkari, D., Hollinshead, M., Roffman, J. L., Smoller, J. W., Zöllei, L., Polimeni, J. R., Fischl, B., Liu, H., & Buckner, R. L. (2011). The organization of the human cerebral cortex estimated by intrinsic functional connectivity. *Journal of Neurophysiology*, *106*(3), 1125–1165. https://doi.org/10.1152/jn.00338.2011
